# Cancer mortality in Europe in 2020, and an overview of trends since 1990

**DOI:** 10.1097/CEJ.0000000000000981

**Published:** 2025-06-26

**Authors:** Claudia Santucci, Paola Bertuccio, Silvia Mignozzi, Margherita Pizzato, Giovanni Corso, Anna Odone, Eva Negri, Carlo La Vecchia, Gianfranco Alicandro

**Affiliations:** a Department of Clinical Sciences and Community Health, University of Milan; b Fondazione IRCCS Ca’ Granda, Ospedale Maggiore Policlinico, Milan; c Department of Public Health, Experimental and Forensic Medicine, University of Pavia, Pavia; d Department of Medical and Surgical Sciences, University of Bologna, Bologna; e Division of Breast Surgery, European Institute of Oncology IRCCS; f Department of Oncology and Hemato-Oncology, University of Milan, Milan; g Medical Direction, Fondazione IRCCS Policlinico San Matteo, Pavia; h Department of Pathophysiology and Transplantation, University of Milan; i Cystic Fibrosis Centre, Fondazione IRCCS Ca’ Granda, Ospedale Maggiore Policlinico, Milan, Italy

**Keywords:** cancer, Europe, mortality, trends

## Abstract

**Introduction:**

Cancer remains a leading cause of death in Europe, with over 1.2 million deaths recorded in the EU-27 in 2020.

**Methods:**

Using WHO death certification data for 33 European countries from 1990 to 2020, we analyzed mortality trends for all neoplasms and 24 cancer sites, stratified by sex and age. We computed age-standardized mortality rates (ASMR) and applied joinpoint regression models to evaluate temporal trends.

**Results:**

In 2020, the leading causes of cancer death in the EU-27 were lung (ASMR: 30.1/100 000), colorectal (14.6/100 000), and prostate (9.8/100 000) cancer in males and breast (13.8/100 000), lung (13.2/100 000), and colorectal (8.6/100 000) cancer in females. Pancreatic cancer was the fourth most common cause of cancer death in both sexes (ASMR: 8.2/100 000 males and 5.8/100 000 females). Most Central and Eastern European countries reported rates over two-fold higher compared to Western Europe. While overall cancer mortality declined since 1990 (average annual percent change: −1.3% in males and −0.8% in females in the EU-27), mortality from pancreatic (+0.2% in males and +0.8% in females) and female lung cancer (+1.9%) increased.

**Conclusion:**

Declines in cancer mortality are attributable to reduced tobacco use, and improvements in organized screening programs and treatment. Pancreatic cancer mortality remains stable, while female lung cancer mortality continues to rise in some countries, largely due to later adoption of smoking and low cessation rates. Lower participation in screening programs and limited access to novel therapies in many Central and Eastern European countries contribute to poorer cancer outcomes, highlighting the need for equitable prevention, early detection, and treatment strategies across Europe.

## Introduction

Cancer remains the second leading cause of death in Europe and the primary cause of premature mortality. Cancer mortality peaked in the late 1980s and has since declined in both Europe and North America (Siegel *et al*., 2025). In 2015, the mortality rate from all cancers in the European Union (EU) was 137.5/100 000 in males and 85.7/100 000 in females, with annual declines of 1.5% since 2006 and 0.8% since 2007, respectively (Bertuccio *et al*., 2019). These downward trends started earlier and were more pronounced in Western European countries, while only modest declines began in most Central and Eastern European countries in the late 1990s.

However, two major cancers—pancreatic cancer in both sexes and lung cancer among females—have shown no improvement in most European countries. These trends reflect differences in exposure to major risk factors, such as tobacco smoking, alcohol consumption, overweight, and obesity (GBD Tobacco Collaborators, 2021; GBD Alcohol Collaborators, 2022; World Health Organization Regional Office for Europe, 2022), as well as disparities in access to screening, early diagnosis, and effective treatment (WHO Regional Office for Europe, 2024). Marked inequalities in lifestyle, prevention strategies, and healthcare access likely continue to contribute to persistent disparities in cancer mortality across Europe (Santucci *et al*., 2022; OECD, 2024).

We provide here an updated and comprehensive report of cancer mortality across Europe from 1990 to 2020.

## Material and methods

We retrieved death certificate data from the WHO database (World Health Organization Statistical Information and System, 2025) for 24 cancer sites and all neoplasms in 33 European countries, covering the period from 1990 to the most recent available year (2020 to 2022). Resident population data were obtained from the United Nations database (United Nations, Department of Economic *et al*., 2024). We analyzed countries with high data quality, except Bulgaria, Greece, North Macedonia, and Poland, whose data were rated as medium quality according to WHO (World Health Organization, 2020). Supplementary Table 1, Supplemental digital content 1, https://links.lww.com/EJCP/A559 lists the 33 included countries, along with the available periods, the mid-year resident population in 2020, and death certification quality.

For each cancer site, country, and calendar year, we computed sex- and age-specific mortality rates for 5-year age groups, from 0–4 to 85+ years. We then derived age-standardized mortality rates (ASMRs) per 100 000 person-years at all ages and in the 35–64 age group, using the Segi’s world standard population.

For the 27 EU member states (EU-27) as a whole and a subset of 23 selected countries with populations over 5 million in 2020, we performed joinpoint regression analysis (Kim *et al*., 2000, 2022) on mortality data for all neoplasms and selected major cancers (i.e. colorectal, pancreatic, lung, breast, and prostate) to identify significant changes in the linear slope (on a logarithmic scale) of the ASMRs over the period considered. The estimated annual percent change for each identified linear segment and the weighted average annual percent change (AAPC) over all available calendar periods as a summary measure were calculated from each joinpoint model.

Ethics committee approval was not required as only anonymous public data were used. Statistical analyses were performed using the R Statistical Software (version 4.3.2; R Development Core Team 2024, Vienna, Austria), SAS version 9.4 (SAS Institute Inc., Cary, North Carolina, USA), and Joinpoint Regression Software, version 5.4.0. April 2025 (Statistical Research and Applications Branch, National Cancer Institute, USA).

## Results

Table 1 shows the ASMRs for all neoplasms and for selected cancer sites per 100 000 males and females of all ages, along with the number of deaths observed in 2020 in 33 European countries and the EU-27. Among males in the EU-27, 671 965 cancer deaths were observed in 2020, corresponding to an overall cancer ASMR of 125.3 per 100 000.

**Table 1:** Age-standardized mortality rates (first row) and number of deaths (second row) from selected cancers per 100 000 males in 33 European countries and the EU-27 in 2020^a^

	Oral cavity/pharynx	Esophagus	Stomach	Colorectum	Liver	Gallbladder	Pancreas	Larynx	Lung	Bone	Connective/soft tissue sarcomas	Skin	Prostate	Testis	Bladder	Kidney	Thyroid	HL	NHL	MM	Leukemias	All neoplasms
ICD-10	C00–C14	C15	C16	C17–C21, C26	C22.0–C22.7	C23–C24	C25	C32	C33–C34	C40–C41	C47, C49	C43–C44	C61	C62	C67	C64–C66, C68	C73	C81	C82–C85, C96	C88, C90	C91–C95	C00–D48
Austria	4.26	3.64	4.08	11.89	5.57	1.46	8.96	1.27	24.54	0.59	0.87	2.80	10.47	0.21	3.10	4.00	0.30	0.30	3.06	1.50	4.01	110.83
	389	332	438	1319	561	154	934	126	2412	29	66	314	1398	16	383	449	36	26	339	181	457	11 769
Belarus	12.84	5.98	15.75	18.16	3.58	1.10	8.63	4.74	40.37	0.40	0.89	2.57	12.89	0.28	4.24	5.91	0.12	0.49	3.13	1.52	4.68	166.96
	840	395	1064	1248	236	73	583	310	2740	21	48	173	907	17	302	398	8	30	208	104	298	11 221
Belgium	3.54	4.50	3.23	10.97	3.73	0.40	6.87	0.94	27.72	0.54	0.76	1.92	8.75	0.18	3.59	3.60	0.15	0.14	2.78	1.54	3.94	107.63
	408	565	430	1578	488	57	893	121	3678	49	80	251	1581	17	581	509	19	22	414	242	565	14 887
Bulgaria	4.69	2.77	8.72	20.43	3.02	0.69	9.70	5.13	36.37	0.67	0.54	2.40	12.81	0.88	5.83	4.28	0.38	0.30	2.25	1.07	3.75	144.31
	305	186	655	1603	209	52	689	351	2578	39	33	182	1194	40	488	309	29	20	148	74	251	10 638
Croatia	7.04	3.67	8.60	26.10	4.38	2.06	8.32	3.19	43.57	0.74	1.09	4.24	13.80	0.47	6.53	5.31	0.36	0.19	3.41	2.07	4.72	167.69
	269	154	395	1272	199	106	390	138	1918	33	31	190	785	12	338	241	14	8	154	102	216	7745
Czech Republic	6.28	4.53	5.07	18.35	3.15	2.47	10.56	1.74	29.13	0.44	0.97	3.15	11.47	0.39	5.04	5.90	0.22	0.24	2.81	2.14	4.75	137.39
	612	477	582	2154	364	302	1181	188	3421	37	82	375	1524	29	642	676	24	27	336	247	561	15 901
Denmark	3.65	4.55	4.55	13.18	3.81	0.85	8.40	0.78	23.19	0.15	0.80	2.56	15.24	0.28	3.58	2.98	0.24	0.17	2.33	1.84	3.68	116.21
	227	305	309	973	255	60	602	53	1691	7	41	184	1358	11	306	208	14	14	180	153	281	8598
Estonia	7.79	5.85	11.76	16.25	5.19	1.40	9.24	2.06	38.35	0.46	1.34	2.96	16.09	0.08	5.76	6.48	–	0.09	3.56	2.03	5.27	161.60
	80	61	140	230	61	17	112	25	467	5	13	38	241	1	84	83	–	1	46	27	61	2029
Finland	2.37	3.83	3.52	10.92	5.48	1.78	8.85	0.51	19.47	0.36	1.12	2.46	10.33	0.26	2.63	3.30	0.49	0.13	3.90	2.00	2.62	99.02
	148	246	235	792	389	121	630	37	1413	14	55	182	920	8	216	239	29	7	293	161	206	7154
France	4.19	3.78	3.52	11.88	6.44	0.49	8.05	1.02	29.14	0.58	0.78	1.93	8.12	0.16	4.27	4.55	0.17	0.17	3.08	1.66	3.93	118.74
	2658	2679	2590	10 062	4804	429	6120	708	20 765	326	496	1510	8863	68	4035	3794	134	114	2651	1595	3369	93 603
Germany	4.37	4.69	4.71	12.63	4.71	1.45	8.66	1.06	26.71	0.42	0.90	2.06	10.70	0.32	2.90	5.27	0.27	0.18	3.21	1.54	4.11	116.01
	3955	4556	5032	14 397	5065	1738	9448	1070	27 751	257	838	2395	15 403	197	3942	6401	295	185	3904	1882	4784	129 916
Greece	2.19	1.73	5.58	11.88	3.04	0.72	8.38	2.52	40.81	0.46	1.20	1.39	8.07	0.22	6.38	3.11	0.38	0.37	2.47	1.76	4.65	128.84
	253	191	763	1890	394	95	1099	324	5366	61	121	202	1785	18	1076	446	42	44	324	281	699	18 439
Hungary	11.84	4.51	7.88	29.60	1.91	2.22	11.51	5.30	52.77	0.31	1.00	3.13	12.29	0.60	6.81	5.38	0.35	0.21	3.28	1.29	4.40	185.61
	954	391	750	2851	173	210	1045	439	4818	21	69	307	1354	38	696	496	32	19	302	127	412	17 209
Iceland	1.24	4.33	2.77	10.80	2.46	0.99	6.13	0.48	14.36	0.20	0.80	2.52	15.21	–	3.44	3.12	0.17	–	3.16	1.52	1.66	87.55
	4	13	10	36	8	4	22	2	56	1	3	11	64	–	16	12	1	–	13	6	7	329
Ireland	3.01	6.89	3.97	13.94	5.42	0.41	6.57	1.16	23.14	0.36	0.80	4.17	11.08	0.16	3.21	3.82	0.19	0.24	3.08	2.03	3.47	110.17
	126	298	190	668	251	23	305	51	1092	13	32	198	613	5	168	183	10	9	159	101	166	5253
Italy	2.85	1.81	5.79	12.82	4.73	1.70	7.79	1.53	26.05	0.51	0.93	2.34	6.76	0.23	4.42	3.82	0.29	0.34	3.17	1.91	4.21	112.11
	2050	1354	5082	11 599	3698	1516	6314	1288	22 188	256	575	1981	7899	88	4713	3379	222	242	2659	1813	3527	97 867
Latvia	8.87	6.30	12.87	17.17	4.68	0.58	10.28	5.03	42.02	0.57	1.06	3.26	20.85	0.58	9.21	6.33	0.21	0.09	3.30	2.20	4.45	178.92
	136	98	223	324	75	9	180	86	725	8	15	58	428	5	174	113	4	1	62	40	75	3147
Lithuania	13.50	7.97	14.10	17.51	5.34	1.29	9.25	4.50	36.96	0.77	0.96	3.43	17.00	0.27	6.51	6.19	0.36	0.25	3.03	2.13	5.62	176.73
	298	187	349	491	129	35	234	119	950	15	21	89	537	4	195	162	8	6	78	56	148	4601
Luxembourg	2.04	3.79	4.89	9.11	3.89	0.61	5.18	0.44	18.77	0.80	0.82	2.99	9.13	0.25	2.92	2.44	0.47	–	1.94	0.95	3.10	85.53
	11	22	27	58	22	4	33	3	112	3	6	19	65	1	19	15	3	–	13	7	22	529
Malta	3.13	2.41	5.73	12.19	5.21	0.32	10.23	0.54	25.85	0.65	0.45	1.89	6.57	0.36	4.96	2.58	0.21	0.36	2.49	1.17	4.12	104.68
	14	13	31	70	25	2	48	3	132	2	2	9	40	1	27	13	1	1	13	7	21	539
Netherlands	2.34	7.16	3.23	13.53	2.69	0.94	6.66	0.78	25.89	0.40	1.02	2.70	11.12	0.21	3.74	4.72	0.26	0.20	3.42	2.01	3.72	113.60
	443	1486	712	3013	572	202	1448	163	5681	54	184	548	3006	21	953	1077	54	40	745	482	837	25 341
North Macedonia	2.95	1.35	8.83	15.43	6.18	1.17	5.66	3.03	39.66	0.86	0.30	3.80	12.29	0.35	4.81	2.29	0.24	0.91	1.71	0.57	2.39	129.61
	51	24	159	286	110	22	104	56	701	15	5	68	243	5	93	38	4	12	27	10	40	2326
Norway	2.44	3.27	3.55	15.42	3.41	0.74	6.81	0.38	22.01	0.37	0.93	3.88	14.28	0.06	3.69	3.46	0.22	0.26	2.59	2.55	3.64	105.60
	120	167	190	849	160	37	364	22	1191	14	41	202	957	3	237	190	12	12	143	142	196	5876
Poland	7.01	3.66	8.58	19.88	1.91	1.61	6.96	4.09	39.01	0.56	1.00	3.68	13.64	0.57	7.97	4.39	0.34	0.34	2.84	1.87	4.22	160.80
	2253	1227	3115	7503	664	592	2431	1409	14 229	167	277	1432	5748	137	3202	1600	125	102	987	707	1507	59 008
Portugal	7.22	4.80	11.33	18.20	7.18	1.29	7.34	2.87	29.25	1.01	1.04	2.07	11.18	0.31	4.28	3.69	0.32	0.40	3.72	2.14	4.59	140.65
	682	477	1367	2432	788	179	894	297	3272	70	99	258	1903	20	652	477	39	35	467	294	541	17 176
Romania	11.15	3.77	10.86	19.73	3.24	1.29	8.73	5.31	41.90	1.44	0.71	2.82	10.09	0.45	6.02	3.67	0.27	0.34	2.37	1.12	3.88	162.18
	1771	625	2015	3858	582	247	1584	886	7349	222	113	538	2345	59	1267	654	47	52	414	215	667	29 479
Serbia	6.44	2.67	6.82	19.88	5.04	1.41	8.46	4.07	47.45	1.06	0.96	2.86	11.27	0.88	6.64	3.56	0.51	0.54	2.88	1.83	4.85	160.82
	431	189	520	1548	380	111	625	302	3407	61	60	220	1049	34	560	258	36	25	192	140	335	11 977
Slovakia	10.95	5.16	7.88	26.45	3.22	2.13	10.31	2.94	32.23	0.87	1.27	3.54	14.17	0.96	6.11	6.74	0.26	0.45	3.95	2.31	4.66	160.53
	492	239	389	1327	159	107	497	142	1602	32	49	176	762	29	319	334	13	21	188	116	237	7876
Slovenia	6.78	4.44	7.32	15.84	8.88	2.10	8.41	2.07	31.46	0.72	0.71	3.74	14.94	0.30	6.49	3.69	0.36	0.37	5.08	2.12	4.03	142.60
	139	97	194	412	211	54	209	49	740	10	11	97	445	5	180	104	8	8	144	57	103	3587
Spain	3.53	2.94	5.19	15.28	5.80	0.86	7.21	2.09	31.45	0.57	0.88	1.74	7.58	0.14	5.16	4.46	0.24	0.26	2.74	1.56	3.40	117.71
	1708	1461	2946	9430	3027	553	3824	1084	16 615	182	368	1078	5922	44	3593	2604	142	139	1555	1004	1902	67 247
Sweden	1.77	2.72	2.66	11.58	2.88	1.53	7.95	0.34	12.42	0.55	0.83	2.99	13.04	0.12	2.81	3.01	0.24	0.13	2.52	2.08	3.26	89.17
	218	347	334	1527	361	217	1012	43	1708	35	92	382	2252	11	475	413	29	14	359	295	454	12 378
Switzerland	3.31	3.59	3.22	9.51	4.30	1.12	6.87	0.69	19.00	0.44	0.92	2.20	9.92	0.26	3.59	2.50	0.23	0.18	2.66	1.88	2.90	90.05
	291	341	312	1012	428	113	683	67	1852	25	62	259	1326	17	442	274	23	18	311	223	316	9497
United Kingdom	3.41	7.56	3.29	14.45	4.64	0.55	6.60	0.89	22.37	0.33	0.96	2.53	12.11	0.13	3.92	3.81	0.20	0.24	3.47	2.05	3.39	113.16
	2244	5667	2615	11 580	3536	462	5042	677	17 796	148	588	2077	12 275	58	3852	2974	160	180	2845	1826	2724	91 923
EU-27	4.73	3.81	5.60	14.64	4.60	1.26	8.16	1.94	30.07	0.56	0.91	2.36	9.83	0.30	4.55	4.49	0.27	0.25	3.06	1.73	4.03	125.28
	20 600	18 083	29 280	81 835	23 521	7087	42 165	9209	152 677	1948	3768	12 995	68 368	887	28 730	24 989	1377	1159	16 934	10 264	22 070	671 965

HL, Hodgkin lymphoma; ICD-10, International Classification of Diseases 10^th^ Revision; MM, multiple myeloma; NHL, non-Hodgkin lymphoma.

a Available year for Portugal and Romania: 2019; for Belarus: 2018; for Malta: 2017; for Norway: 2016.

Table 2 gives the corresponding figures for females. The overall female cancer mortality rate in the EU-27 was 80.0/100 000, corresponding to 537 867 deaths observed in 2020.

**Table 2:** Age-standardized mortality rates (first row) and number of deaths (second row) from selected cancers per 100 000 females in 33 European countries and the EU-27 in 2020^a^

	Oral cavity/pharynx	Esophagus	Stomach	Colorectum	Liver	Gallbladder	Pancreas	Larynx	Lung	Bone	Connective/soft tissue sarcomas	Skin	Breast	Uterus	Ovary	Bladder	Kidney	Thyroid	HL	NHL	MM	Leukemias	All neoplasms
ICD-10	C00–C14	C15	C16	C17–C21, C26	C22.0–C22.7	C23–C24	C25	C32	C33–C34	C40–C41	C47, C49	C43–C44	C50	C53–C55	C56–C57.4	C67	C64–C66, C68	C73	C81	C82–C85, C96	C88, C90	C91–C95	C00–D48
Austria	1.12	0.78	2.32	6.58	1.50	0.97	6.76	0.17	14.26	0.41	0.64	1.52	12.87	3.59	4.23	0.99	1.51	0.26	0.16	1.80	0.98	2.43	75.37
	140	92	315	967	193	142	929	20	1635	26	72	237	1646	435	525	172	252	49	17	275	151	394	10 034
Belarus	1.12	0.38	5.86	9.99	0.82	1.02	3.62	0.16	3.40	0.23	0.52	1.59	11.40	7.04	4.71	0.36	1.56	0.22	0.36	1.72	1.06	2.56	69.73
	119	40	679	1240	93	121	433	15	382	17	53	185	1138	695	469	54	192	27	26	183	120	283	7626
Belgium	0.79	1.06	1.38	7.13	1.52	0.45	5.45	0.18	14.38	0.19	0.69	1.11	13.30	3.54	3.71	0.83	1.67	0.22	0.06	1.44	1.09	2.26	73.63
	125	183	251	1444	263	83	961	25	1997	28	88	194	2058	522	592	197	304	46	13	304	223	438	12 336
Bulgaria	1.08	0.43	4.03	10.39	1.24	0.68	5.60	0.28	10.41	0.34	0.33	1.20	15.13	9.52	5.54	1.32	1.37	0.26	0.26	1.44	0.80	2.08	85.08
	97	36	424	1145	124	74	559	22	886	30	20	135	1408	754	465	139	123	32	17	145	79	178	7889
Croatia	1.16	0.65	4.08	12.29	1.45	2.04	5.57	0.22	16.47	0.61	0.56	2.25	13.06	5.92	6.28	1.74	1.89	0.12	0.27	2.16	1.15	2.24	93.55
	67	36	270	875	99	135	382	13	901	24	21	156	722	319	333	136	134	11	12	140	86	174	5763
Czech Republic	1.56	0.77	2.85	9.52	1.19	2.16	7.09	0.15	12.80	0.27	0.60	1.58	12.27	5.50	5.41	1.34	2.52	0.21	0.26	1.63	1.28	2.57	85.91
	191	100	392	1529	176	360	1146	21	1883	30	65	258	1710	720	691	253	408	36	29	275	207	419	12 815
Denmark	1.52	1.36	1.46	9.15	1.67	1.16	6.47	0.12	20.90	0.27	1.06	1.67	13.23	3.73	4.34	1.44	1.34	0.27	0.14	1.21	0.89	2.34	90.82
	112	111	111	846	128	104	547	8	1689	13	51	140	1047	270	334	140	127	23	15	124	103	218	7555
Estonia	1.06	0.67	5.36	7.84	2.06	1.26	6.09	0.22	6.31	0.31	1.35	1.60	13.04	7.18	6.08	0.75	2.79	0.42	0.05	2.08	2.04	2.81	81.85
	17	15	121	215	43	25	148	2	138	3	18	38	257	120	101	23	70	10	1	53	51	62	1772
Finland	0.82	1.25	2.15	7.20	1.80	1.54	7.00	0.05	10.14	0.19	0.64	1.12	12.46	2.99	4.39	0.76	1.72	0.40	0.16	2.36	1.16	1.19	69.45
	77	110	177	705	174	151	682	3	869	6	42	103	946	262	382	94	173	38	8	237	141	129	6257
France	1.02	0.87	1.45	7.59	1.84	0.42	5.61	0.12	12.08	0.32	0.65	1.06	13.86	3.70	3.49	0.88	1.37	0.17	0.07	1.61	1.01	2.26	72.35
	887	886	1479	9227	1948	572	6086	97	9796	238	484	1125	12 534	3455	3314	1248	1698	214	62	2089	1390	2679	73 719
Germany	1.15	1.10	2.40	7.62	1.84	1.31	6.36	0.16	14.86	0.26	0.71	1.11	14.61	3.80	4.41	1.04	2.09	0.24	0.09	1.87	0.94	2.28	80.63
	1397	1398	3321	11 951	2419	2102	9474	198	17 066	183	725	1593	18 425	4304	5453	1935	3515	396	132	3098	1530	3573	109 636
Greece	0.84	0.27	2.41	6.96	1.15	0.37	4.80	0.25	11.07	0.27	0.74	1.06	13.27	4.20	4.16	0.98	1.00	0.20	0.15	1.57	1.21	2.47	73.05
	146	48	418	1419	189	82	901	35	1648	41	92	189	2191	591	543	225	202	40	19	254	253	473	12 391
Hungary	2.24	0.82	3.80	14.52	0.75	2.04	7.59	0.67	26.76	0.36	0.83	1.87	16.76	7.55	5.62	2.06	2.34	0.37	0.12	1.93	1.07	2.95	113.88
	259	103	550	2211	105	298	1123	72	3341	26	70	271	2195	873	702	333	333	51	15	270	152	403	15 242
Iceland	0.45	1.09	1.64	10.64	0.60	1.89	5.00	0.29	17.63	–	0.68	1.18	13.29	4.75	3.49	2.14	1.91	0.35	–	1.63	0.95	1.48	78.71
	3	5	7	40	4	6	21	1	66	–	2	4	43	16	13	10	9	1	–	8	5	8	302
Ireland	1.01	2.47	2.06	8.85	2.74	0.68	5.30	0.24	17.06	0.28	0.82	1.19	15.62	4.77	5.72	1.11	1.50	0.17	0.14	1.85	1.34	1.84	86.50
	56	142	105	509	149	41	301	12	886	11	30	66	771	230	291	77	87	12	8	117	87	103	4620
Italy	1.01	0.52	3.03	8.08	1.49	1.37	5.88	0.20	10.61	0.27	0.58	1.20	14.12	3.64	3.98	0.97	1.32	0.26	0.20	1.88	1.27	2.46	75.41
	1061	548	3550	9979	1751	1692	6635	188	10 110	198	460	1225	13 124	3134	3394	1385	1570	309	175	2177	1600	2725	79 991
Latvia	1.83	0.96	5.32	10.55	1.25	0.83	6.56	0.26	7.64	0.67	0.46	1.67	16.68	8.85	8.80	0.99	2.92	0.46	0.34	1.97	2.10	2.67	97.15
	53	23	182	379	38	29	245	6	218	9	15	77	451	237	213	48	105	18	6	68	59	91	2976
Lithuania	1.50	0.90	5.91	8.50	1.46	1.56	5.42	0.07	6.47	0.37	0.53	1.68	14.46	10.08	7.45	0.94	1.99	0.46	0.08	1.61	1.30	2.22	87.42
	54	32	261	463	62	67	285	2	279	8	23	78	560	365	273	61	103	21	3	82	63	129	3823
Luxembourg	1.26	0.33	0.97	7.43	1.29	0.51	6.31	0.11	12.95	–	0.43	1.19	12.63	1.91	2.79	0.80	0.70	0.07	0.17	1.83	0.63	1.97	64.24
	7	2	10	67	8	4	45	1	85	–	3	11	95	13	23	10	8	1	1	13	5	20	492
Malta	1.95	0.95	2.04	12.00	0.85	0.62	4.91	0.18	9.93	0.66	0.58	0.79	12.35	3.58	6.62	1.41	1.07	–	0.20	2.25	0.66	1.98	72.94
	12	7	13	76	4	6	35	2	49	2	3	5	64	20	32	8	8	–	1	12	7	13	424
Netherlands	1.01	2.04	1.86	10.66	1.65	0.93	5.85	0.18	20.12	0.27	0.67	1.75	14.29	3.57	4.48	1.34	2.02	0.22	0.12	1.96	1.31	2.05	89.57
	247	527	438	2903	384	250	1494	42	4399	41	113	413	3061	790	1041	406	560	67	28	560	382	548	21 748
North Macedonia	0.65	0.26	4.01	9.04	3.99	0.55	4.17	0.52	9.37	0.48	0.03	2.08	17.27	6.97	3.44	1.45	0.74	0.30	0.60	1.10	0.75	1.72	79.38
	15	5	85	196	87	13	87	11	188	10	1	43	333	131	64	30	14	6	11	22	16	33	1582
Norway	0.93	0.84	1.51	12.49	1.40	0.66	5.26	0.13	17.26	0.22	0.41	2.21	11.23	4.30	5.71	1.09	1.50	0.28	0.09	1.67	1.24	2.34	82.47
	64	58	105	900	78	43	346	7	1043	13	24	150	623	246	314	93	108	23	7	118	92	142	5250
Poland	1.67	0.77	3.26	10.19	0.87	1.87	5.00	0.47	16.85	0.37	0.66	1.98	14.82	7.37	6.27	1.51	1.91	0.39	0.16	1.56	1.35	2.44	98.19
	764	355	1657	5649	445	988	2542	195	8009	145	244	1401	6956	3496	2708	915	1052	222	65	817	783	1265	49 694
Portugal	0.91	0.41	4.76	9.49	1.64	0.97	4.05	0.12	7.98	0.47	0.89	1.16	13.06	4.93	2.70	0.91	1.23	0.25	0.14	2.10	1.43	2.57	72.15
	147	75	882	1783	281	190	759	15	1133	44	100	219	1880	715	410	215	228	59	23	397	286	398	12 000
Romania	1.29	0.51	3.88	9.49	1.34	0.91	5.13	0.30	10.82	0.68	0.44	1.46	14.74	11.26	4.83	1.23	1.31	0.41	0.21	1.50	0.79	2.37	87.44
	286	114	1043	2636	359	250	1363	67	2454	145	90	391	3374	2246	1040	395	338	105	36	313	202	545	20 828
Serbia	1.46	0.52	3.04	10.40	2.77	1.33	5.39	0.40	19.36	0.53	0.67	1.77	20.24	10.29	4.69	1.55	1.72	0.33	0.26	1.93	1.20	2.93	106.47
	126	48	276	1005	266	144	544	39	1603	42	46	187	1782	782	381	158	162	36	20	179	115	236	9415
Slovakia	1.68	0.54	3.59	12.22	1.33	2.60	6.66	0.28	10.70	0.31	0.61	1.79	15.51	7.63	5.70	1.29	2.05	0.38	0.25	2.80	1.65	2.87	92.41
	101	37	249	890	91	205	481	18	700	17	36	123	1067	476	347	105	160	29	18	190	126	207	6313
Slovenia	0.63	0.51	3.40	8.17	2.50	1.41	6.31	0.13	15.45	0.21	0.28	3.02	15.16	4.44	4.08	1.29	1.96	0.26	0.33	2.74	1.30	2.07	85.33
	20	19	120	296	90	59	210	4	448	5	10	101	472	139	116	64	75	11	7	119	59	86	2849
Spain	1.01	0.55	2.60	8.03	1.79	0.69	4.83	0.16	9.52	0.34	0.63	0.88	10.45	3.58	3.56	0.80	1.40	0.26	0.20	1.56	0.98	2.07	64.79
	691	362	1971	6740	1390	643	3603	97	5303	146	321	739	6572	2273	2072	830	1100	217	95	1270	885	1461	45 494
Sweden	0.76	1.05	1.31	9.33	1.13	2.03	6.18	0.08	12.19	0.22	0.88	1.81	10.60	3.46	4.11	0.93	1.53	0.29	0.07	1.53	1.31	1.89	73.25
	120	144	183	1541	161	327	948	8	1849	15	99	244	1389	483	558	173	253	42	12	284	229	308	11 196
Switzerland	1.33	0.91	1.70	6.29	1.53	1.02	5.39	0.06	11.81	0.24	0.66	1.11	11.50	2.41	3.84	1.32	1.20	0.13	0.05	1.49	1.13	2.02	64.88
	144	123	201	850	182	152	701	7	1346	20	64	157	1305	281	436	174	162	23	10	240	168	252	8009
United Kingdom	1.29	2.35	1.49	9.99	2.47	0.75	5.24	0.20	17.46	0.26	0.67	1.41	14.14	4.32	5.01	1.55	1.90	0.24	0.12	2.04	1.29	2.03	87.89
	1077	2329	1381	9935	2267	755	4906	178	15 853	130	454	1329	11 547	3434	4132	1793	1886	228	120	2159	1447	1881	80 378
EU-27	1.15	0.84	2.63	8.59	1.57	1.15	5.77	0.21	13.22	0.32	0.66	1.29	13.79	4.74	4.40	1.08	1.66	0.26	0.15	1.76	1.12	2.32	79.95
	7127	5500	18 490	66 424	11 074	8878	41 903	1172	77 759	1435	3293	9533	85 006	27 250	25 952	9589	12 987	2064	819	13 683	9142	17 039	537 867

HL, Hodgkin lymphoma; ICD-10, International Classification of Diseases 10^th^ Revision; MM, multiple myeloma; NHL, non-Hodgkin lymphoma.

a Available year for Portugal and Romania: 2019; for Belarus: 2018; for Malta: 2017; for Norway: 2016.

**Fig. 1:**
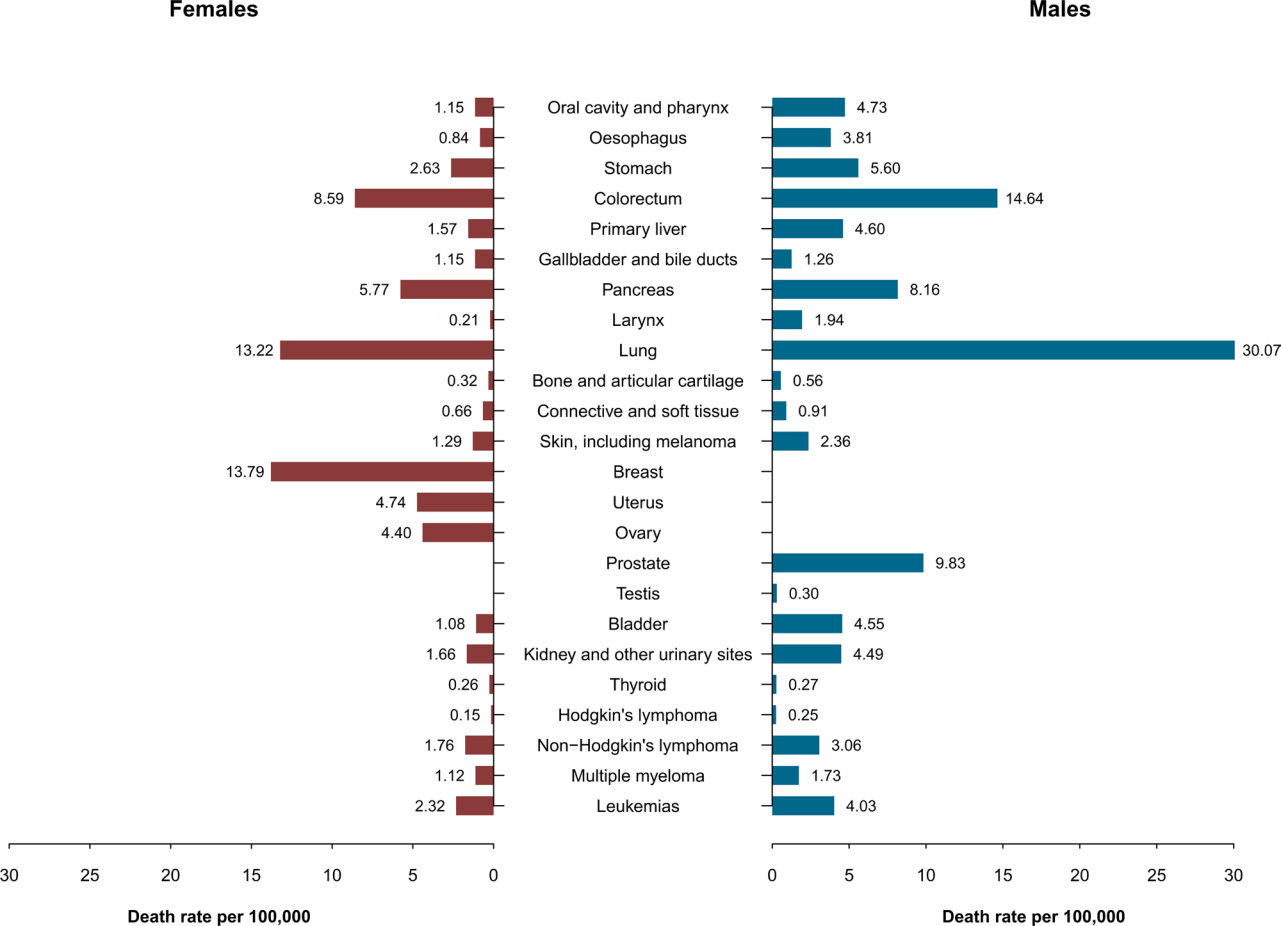
Age-standardized death rates per 100 000 males and females from 24 cancer sites in the EU-27, 2020.

Figure 1 shows the ASMRs in 2020 per 100 000 by sex and cancer site in the EU-27. In the EU-27, lung cancer was the leading cause of cancer death among males at all ages, with an ASMR of 30.1/100 000, followed by colorectal (14.6/100 000), prostate (9.8/100 000), pancreatic (8.2/100 000), and stomach cancer (5.6/100 000). For the other cancer sites considered, the ASMRs were less than 5/100 000 males. Breast cancer was the leading cause of female cancer deaths (ASMR: 13.8/100 000), followed by lung (13.2/100 000), colorectal (8.6/100 000), and pancreatic cancer (5.8/100 000).

**Fig. 2:**
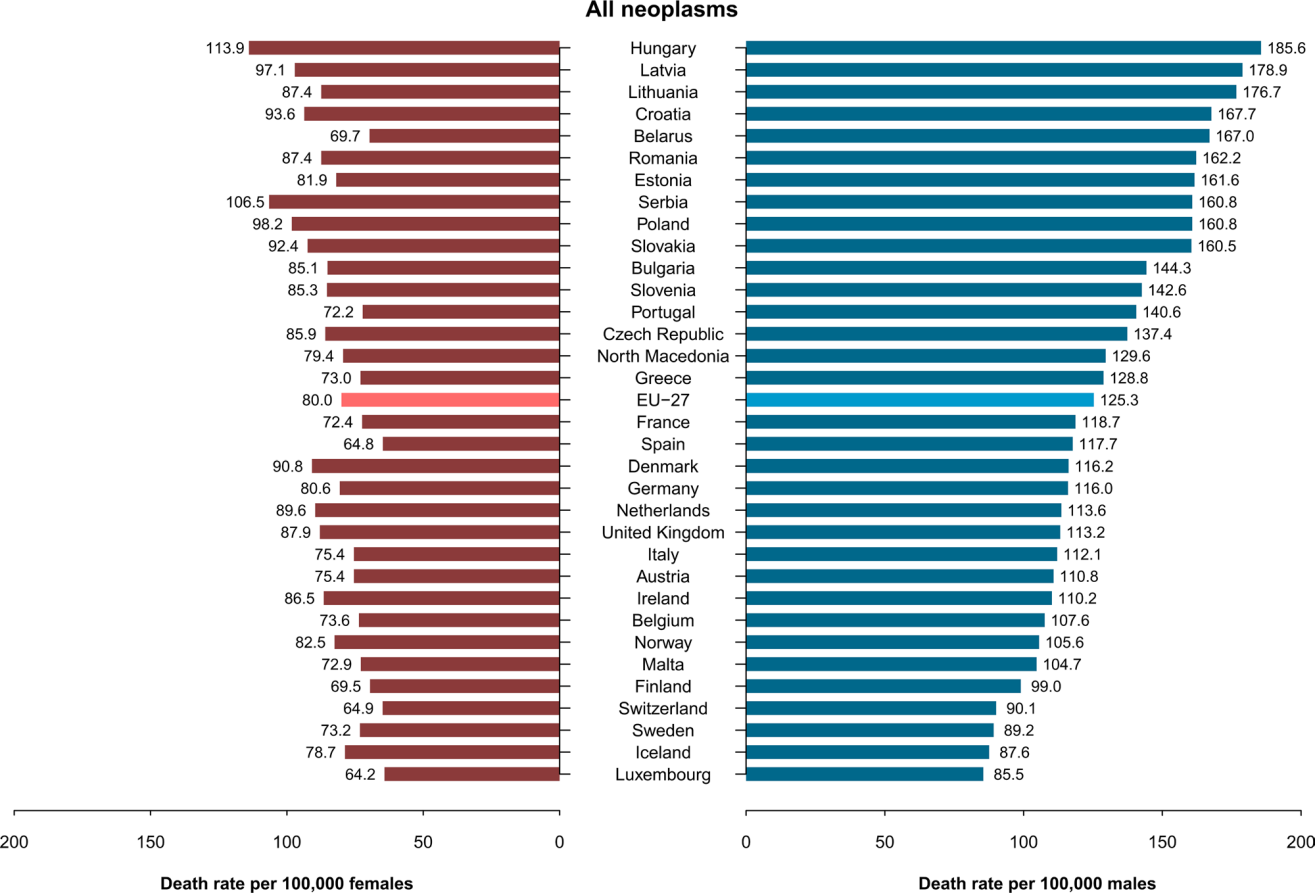
Age-standardized death rates from all neoplasms per 100 000 males and females in 33 European countries and the EU-27 in 2020. For some countries, mortality data for 2020 were not available and the most recent available data were used: 2019 for Portugal and Romania, 2018 for Belarus, 2017 for Malta, and 2016 for Norway.

Figure 2 presents the ASMRs per 100 000 from all neoplasms in 2020, using bar plots ordered by descending male rates in 23 European countries and the EU-27. There was a more than two-fold difference in males and approximately a two-fold difference in females between the highest rates, observed in Hungary (185.6/100 000) and other Central and Eastern European countries, and the lowest rates observed in some Nordic and Southern European countries (e.g. 85.5/100 000 in males and 64.2/100 000 in females in Luxembourg).

Supplementary Figures 1–5, Supplemental digital content 1, https://links.lww.com/EJCP/A559 display the corresponding information for colorectal, pancreatic, lung, breast, and prostate cancer. Among males, colorectal cancer ASMRs ranged from below 10/100 000 in Luxembourg and Switzerland to around 26–30/100 000 in Hungary, Slovakia, and Croatia. Among females, rates ranged from 6.3–6.6/100 000 in Switzerland and Austria to 14.5/100 000 in Hungary.

Pancreatic cancer mortality in males ranged between 7 and 10/100 000 in most countries considered, with higher rates observed in Hungary (11.5/100 000), the Czech Republic (10.6/100 000), Slovakia and Latvia (10.3/100 000). Among females, the ASMRs ranged from around 4/100 000 in Portugal to 7.6/100 000 in Hungary, with comparatively high rates in Central and Eastern Europe.

Lung cancer mortality in males largely varied across Europe, ranging between 12.4/100 000 in Sweden and 52.8/100 000 in Hungary. The highest rates were registered in Eastern European countries, with ASMRs exceeding 35/100 000. Among females, the highest lung cancer mortality rates were recorded in Hungary (26.8/100 000), Denmark (20.9/100 000), the Netherlands (20.1/100 000) and Poland (16.9/100 000), while the lowest ones (3.4–6.5/100 000) were found in the Baltic countries.

Prostate cancer mortality was highest in the Baltic and Scandinavian countries (14–21/100 000), and lowest in Italy and other Southern European countries (7–9/100 000).

Breast cancer mortality also varied considerably, with the highest rates observed in Serbia (20.2/100 000), North Macedonia (17.3/100 000), Hungary (16.8/100 000), and Latvia (16.7/100 000) and the lowest ones recorded in Spain, Sweden and Norway (ASMRs around 10–11/100 000).

In the EU-27, ASMRs in 2020 for the truncated age group (35–64 years) were higher than those observed for all ages for breast, lung, and colorectal cancers (Supplementary Tables 2 and 3, Supplemental digital content 1, https://links.lww.com/EJCP/A559).

**Fig. 3:**
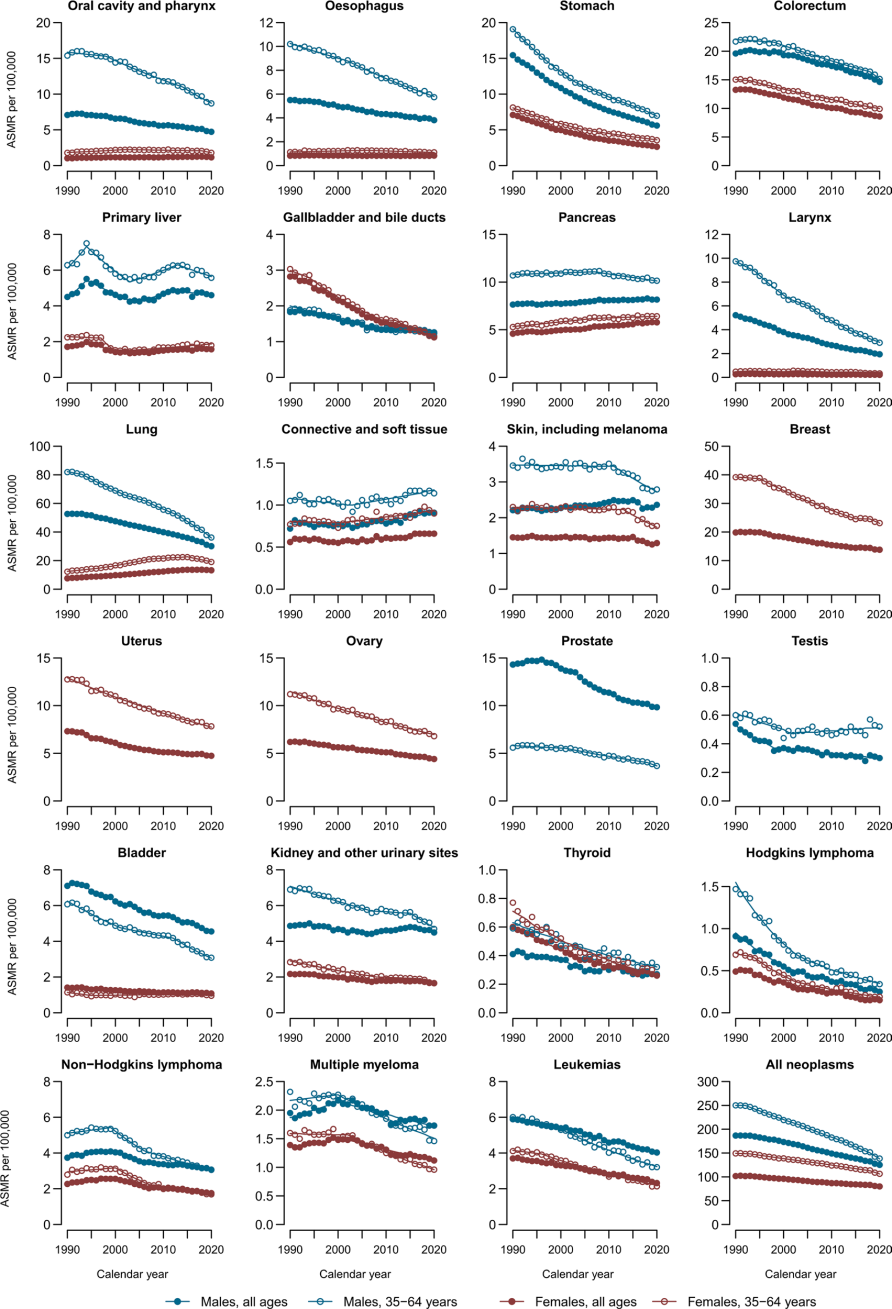
Joinpoint analysis of trends in the age-standardized mortality rates (ASMR) per 100 000 males and females at all ages and in the 35–64 age group from 23 cancer sites and all neoplasms in the EU-27 from 1990 to 2020.

Figure 3 shows the joinpoint analysis for 23 cancer sites and all neoplasms in the EU-27, stratified by sex and age (all ages and the 35–64 age group). Favorable mortality trends were observed for most cancer sites in both sexes since 1990. Exceptions were pancreatic cancer (AAPC: +0.2% for males and +0.8% for females), soft connective tissue sarcomas (+0.6% for males and +0.4% for females), and for females, oral cavity cancer (+0.4%) and lung cancer (+1.9%). Declines in mortality were generally larger in the 35–64 age group than in the overall population.

**Fig. 4:**
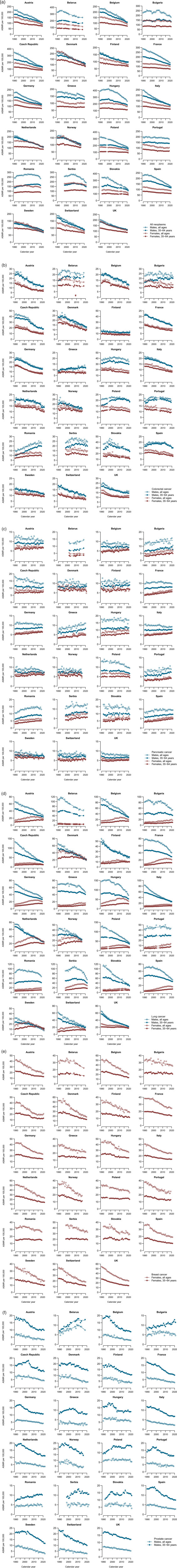
(a) Joinpoint analysis of trends in the age-standardized mortality rates (ASMR) from **all neoplasms** per 100 000 males and females at all ages and in the 35–64 age group in 23 European countries from 1990 to 2022. (b) Joinpoint analysis of trends in the age-standardized mortality rates (ASMR) from **colorectal cancer** per 100 000 males and females at all ages and in the 35–64 age group in 23 European countries from 1990 to 2022. (c) Joinpoint analysis of trends in the age-standardized mortality rates (ASMR) from **pancreatic cancer** per 100 000 males and females at all ages and in the 35–64 age group in 23 European countries from 1990 to 2022. (d) Joinpoint analysis of trends in the age-standardized mortality rates (ASMR) from **lung cancer** per 100 000 males and females at all ages and in the 35–64 age group in 23 European countries from 1990 to 2022. (e) Joinpoint analysis of trends in the age-standardized mortality rates (ASMR) from **breast cancer** per 100 000 females at all ages and in the 35–64 age group in 23 European countries from 1990 to 2022. (f) Joinpoint analysis of trends in the age-standardized mortality rates (ASMR) from **prostate cancer** per 100 000 males at all ages and in the 35–64 age group in 23 European countries from 1990 to 2022.

Figures 4a–f show mortality trends for all neoplasms and main cancer sites in 23 major European countries and the EU-27, stratified by sex and age group. Detailed results from the joinpoint regression analyses are provided in Supplementary Tables 4–10, Supplemental digital content 1, https://links.lww.com/EJCP/A559.

In the EU-27, cancer mortality declined annually since 1990 by 1.3% in males and 0.8% in females across all ages, and by 2% in males aged 35–64 and 1.1% in females aged 35–64. Mortality trends declined in most countries for both sexes and across all age groups, with declines starting after 2000 in Central and Eastern European countries. However, trends remained approximately stable in Bulgaria, Greece, Portugal, Romania and Serbia for both sexes.

In the EU-27, colorectal cancer mortality declined annually by 1% in males and 1.4% in females across all ages, and by 1.2% in males and 1.4% in females aged 35–64. Most major Western European countries showed declining trends, except for the most recent period in the United Kingdom. Trends were less favorable among males in Central and Eastern European countries, including Bulgaria (AAPC: +0.6%), Poland (+0.5%), Romania (+2.5%), and Serbia (+0.5%). Among females, a notable increase in mortality was observed in Romania (+1.1%).

Over the whole period, pancreatic cancer mortality in the EU-27 increased annually by 0.2% in males and 0.8% in females across all ages. In the age group 35–64, AAPCs were: −0.2% in males and +0.6% in females. Mortality rates increased for both sexes in most countries, except in Denmark, Finland, Sweden, Switzerland, the Czech Republic, Poland, and the United Kingdom.

Throughout the entire study period, mortality rates from lung cancer in the EU-27 declined by 1.9% annually among males of all ages, while rates increased by a similar amount among females. In the 35–64 age group, mortality rates declined by 2.7% in males and increased by 1.5% in females. Declining trends in male lung cancer mortality were observed in all countries except Portugal and Romania. Among females, trends in lung cancer ASMR increased significantly in all countries considered (AAPCs ranged from +0.4% in Sweden to +3.4% in Spain), except in Belarus (−1.6%), Denmark (−0.5%) and the United Kingdom (−0.6%). Decreasing trends were, however, observed in recent years in some countries, including Belgium, the Czech Republic, Denmark, Hungary, the Netherlands, Poland, Serbia, Sweden and the United Kingdom.

In the EU-27 breast cancer mortality rates declined by 1.2% across all ages and by 1.8% in the 35–64 age group. Favorable trends were observed in most countries, with the exceptions of limited declines or stable rates in Bulgaria, Poland, Romania, Serbia, and Slovakia. The largest declines were observed in Switzerland (AAPC: −2.5%) and in the United Kingdom (AAPC: −2.3%).

Prostate cancer mortality declined by 1.2% annually in the EU-27 across all ages and by 1.5% in the 35–64 age group. Mortality rates consistently declined in Western Europe over the last two decades. However, some Central and Eastern European countries reported upward trends, including Bulgaria (AAPC: +1.2%), Poland (+1.1%), Romania (+1.3%), Serbia (+0.7%), and Slovakia (+0.9%).

## Discussion

Over the past three decades, cancer mortality in Europe decreased by an average of 1.3% per year in males and 0.8% in females across all ages, with sharper reductions among middle-aged population. Throughout Europe, decreases were observed for colorectal, stomach, breast, and prostate cancers. Lung cancer mortality decreased in males, while it increased or remained stable in females. Pancreatic cancer showed a slight increase over the study period. Despite overall progress, significant disparities persisted, particularly in Central-Eastern Europe, where mortality remained higher than in the other areas. In 2020, the leading causes of cancer death were lung, colorectal, and prostate cancers among males, and breast, lung, and colorectal cancers among females. Pancreatic cancer became the fourth leading cause of cancer death in both sexes.

Several key factors have contributed to the declining trends in cancer mortality in Europe. Smoking remains the major contributor to the cancer burden in Europe, causing more than 750 000 cases annually, with lung cancer accounting for over half of these, and is responsible for approximately 20 to 25% of all cancer deaths (Kulhanova *et al*., 2020; Collatuzzo *et al*., 2024). Smoking cessation efforts, including tobacco taxes, smoking bans, and public health campaigns, appreciably reduced the incidence and mortality of smoking-related cancers across Europe (GBD Tobacco Collaborators, 2021; Willemsen *et al*., 2022). However, these measures remain inadequate in several countries. Additionally, population-based screening programs for breast, cervical, and colorectal cancers were effective in reducing mortality by detecting cancers at earlier, more treatable stages (Gini *et al*., 2020; Jansen *et al*., 2020; Zielonke *et al*., 2020). Advances in chemotherapy, the introduction of targeted therapy, hormone therapies and immunotherapies as well as advances in existing chemotherapy, radiotherapy and surgery improved treatment and survival for several solid and hematological tumors, including breast, with the largest absolute number of avoided deaths, melanoma, colorectal, and prostate cancer, leukemia, Hodgkin lymphoma (HL) and non-HL (Bjorkholm *et al*., 2011; Jacobs *et al*., 2022). Although to a lesser extent, survival also improved for certain difficult‐to‐treat cancers (Wang *et al*., 2025; Warwar *et al*., 2025). Despite notable advances in cancer care, male cancer mortality in Hungary, Latvia, and Lithuania remained approximately twice as high as in Luxembourg, the European country with the lowest rate, and in other countries with similar rates, such as Iceland, Sweden, and Switzerland. Similarly, among females, cancer mortality in Hungary and Serbia was approximately 1.7 times higher than in Luxembourg or Switzerland. These disparities could be attributed to the geographic distribution of cancer risk factors, participation in organized screening programs, and access to effective treatment.

In 2020, the proportion of current smokers aged 15 and older ranged from 6% in Sweden to 49% in Bulgaria (European Commission). For women, smoking prevalence ranged between 8% in Sweden and 38% in Greece. Values above 30% were also observed among males in Greece, Lithuania, Latvia, Croatia, and Romania, as well as among females in Croatia. For two major contributors to cancer mortality—colorectal and breast cancers—there were significant differences in screening participation rates across Europe (European Commission - Eurostat). Colorectal cancer screening rate among eligible individuals ranged between 8.1% in Hungary to 77.3% in Finland. For breast cancer, the percentage of females aged 50–69 who had mammography within the past 2 years ranged from 28.5% in Slovakia to 83% in Denmark. Screening participation was also below 50% in France, Malta, Austria, Poland, Latvia, Hungary, and Cyprus.

Equity in patient access to effective therapies is crucial to achieve better prognosis. However, granting access to all novel cancer treatments is increasingly challenging due to the substantial rise in cancer care expenditure across Europe over the past decade. Consequently, access to innovative cancer therapies through public health systems varies considerably between European countries. Some Central and Eastern European countries have often faced lower access rates than in Northern and Western Europe, both in terms of the availability of novel treatments and the time between European Medicines Agency marketing authorization and national reimbursement approval (OECD, 2020; Hofmarcher *et al*., 2023).

The decline in colorectal cancer mortality in Western Europe is due to earlier diagnosis and improved treatment. However, the reversal of this trend in the United Kingdom warrants attention and can be linked to the obesity epidemic (Mulrenan *et al*., 2023). There were, however, delays in the implementation of innovative and effective therapies in most Central and Eastern European countries for colorectal neoplasms, as well as other highly curable cancers, such as testicular cancer (European Federation of Pharmaceutical Industries and Associations, 2023; OECD, 2024).

Pancreatic cancer remained a leading cause of cancer-related death in Europe, with mortality rates showing no significant decline over the past three decades. Previous research reported rises in pancreatic cancer incidence and mortality, particularly in high-income countries, with a notable increase among individuals over 50 years old and, in some regions, even among younger populations (Huang *et al*., 2021; Yu *et al*., 2025). Currently, there are no effective methods for prevention, early detection, or treatment. Unlike lung or cervical cancer, which are largely attributable to single, well-established risk factors, pancreatic cancer is associated with a wide range of lifestyle, metabolic, and health-related risk factors, each showing only a modest association (Maisonneuve and Lowenfels, 2015). At diagnosis, 80% of patients present with locally advanced or metastatic disease, and among the remaining 20% with localized resectable neoplasm, surgery remains the only potentially curative treatment. However, even after complete surgical resection, up to 80% of patients experience local or distant relapses, likely due to the presence of micrometastatic disease at the time of diagnosis (Michl *et al*., 2021). Although some progress has been made in early detection and therapeutic programs, the prognosis remains poor, with a 5-year survival rate below 10% (Santucci *et al*., 2020).

Lung cancer mortality declined among males but remained stable or increased among females, reflecting historical differences in smoking trends. In 14 countries, lung cancer mortality surpassed female breast cancer mortality, a shift attributable to the delayed epidemic of smoking among females, who adopted the habit in larger numbers only from the 1970s, largely due to shifting social and cultural norms. While smoking prevalence among males began to decline earlier due to increased awareness and public health interventions, a similar decline among females lagged by approximately one to two decades, leading to a subsequent rise in smoking-attributable female mortality (Lortet-Tieulent *et al*., 2015; Janssen *et al*., 2021). Besides smoking, socioeconomic disparities play a role, with high-income regions experiencing a decreasing burden due to better prevention, early detection, and treatment (Pizzato *et al*., 2023). The introduction of lung cancer screening in high-risk populations may contribute to further mortality reductions, particularly by improving early detection and treatment (Zhang *et al*., 2025).

Since the late 1990s, breast cancer mortality has declined substantially, with an average annual reduction of 1.2% across all ages and 1.8% among individuals aged 35–64 years. This notable achievement can be linked to the early detection of localized cancers as well as the availability of a broad range of treatment options, including surgery, radiation therapy, chemotherapy, hormone therapy, targeted therapy, and immunotherapy (Zhai *et al*., 2023).

In the EU-27, cancer deaths under the age of 65 account for about 21–22% of all cancer mortality in both sexes. However, the sharper decline in mortality among younger age groups suggests that their contribution to overall cancer mortality is expected to decrease in the coming years.

HL showed the most pronounced decline in mortality, with an annual reduction of approximately 4%. Although there are no clearly defined major risk factors for the development of HL, familiarity, certain viral exposures, and immune suppression have been associated with increased risk. The significant decline in HL-related mortality is largely attributed to advances in disease management. Optimal management of patients with HL requires accurate diagnosis and careful staging of the disease, allowing for risk-adapted therapy. Patients with early stage disease typically receive combined modality treatment, including abbreviated courses of combination chemotherapy followed by involved-field radiation therapy. In contrast, those with advanced-stage disease are generally treated with longer courses of chemotherapy alone. Recently, novel agents such as brentuximab vedotin and anti-PD-1 antibodies have been included into standard combination regimens. For patients who relapse after initial therapy, high-dose chemotherapy followed by autologous stem cell transplantation remains the standard of care (Ansell, 2022).

Death certification relies on the physician’s clinical judgment and the information available at the time of death, which can lead to misclassification, particularly in patients with multiple comorbidities or when the primary site of metastatic cancer is uncertain. Furthermore, errors or omissions in completing death certificates can result in a significant number of deaths being assigned to less-informative categories—often referred to as ‘garbage codes’—such as ‘unspecified cancer site’. While the overall impact of such misclassification is expected to be modest for major cancer types, it may lead to underestimation of mortality for less common cancers (Monasta *et al*., 2022). Notably, in many countries, a substantial proportion of uterine cancer deaths are recorded as ‘malignant neoplasm of uterus, part unspecified’, which should be taken into account when comparing estimates and trends across countries (Loos *et al*., 2004).

A key strength of this study is the comprehensive evaluation of cancer mortality trends over the past three decades, along with cross-country comparisons within Europe. The study period also includes the first year of the COVID-19 pandemic, during which, as reported in other studies, no measurable impact on cancer mortality was observed (Pizzato *et al*., 2024). By systematically analyzing data across multiple countries and major cancer types, the study highlights regional patterns and disparities in cancer outcomes.

The study found declining trends in cancer mortality across Europe but also highlighted substantial and geographical disparities, with Central and Eastern European countries exhibiting higher mortality rates compared to Western and Northern countries, with a persistent generational gap since the end of nonmarket economies in these regions. To reduce these inequalities, more stringent tobacco control measures, effective implementation of screening programs, and more equitable access to both diagnosis and treatment are urgently needed.

## Acknowledgements

The funder did not play a role in the design of the study; the collection, analysis, and interpretation of the data; the writing of the manuscript; and the decision to submit the manuscript for publication.

Data is derived from a source in the public domain. We retrieved death counts from the WHO database and resident population data from the United Nations Population Division database.

This work was supported by the AIRC Foundation, Italy (grant no. 22987 to E.N.). C.S. and C.L.V. were also supported by the EU funding within the NextGenerationEU-MUR PNRR Extended Partnership initiative (no. PE00000007, INF-ACT). The Department of Pathophysiology and Transplantation is funded by the Italian Ministry of Education and Research - MUR (‘Dipartimenti di Eccellenza’ Programme 2023-27, University of Milan). The funding sources had no role in the design and conduct of the study; collection, management, analysis, and interpretation of the data; preparation, review, or approval of the manuscript; and the decision to submit the manuscript for publication.

C.S.: Data curation, formal analysis, methodology, and writing – original draft. P.B.: Investigation and writing – review & editing. S.M.: Data curation and writing – review & editing. M.P., G.C., A.O., and E.N.: Writing – review & editing. C.L.V.: Investigation and writing – review & editing. G.A.: Writing – original draft.

### Conflicts of interest

There are no conflicts of interest.

## Supplementary Material
