# Supplementary material for: Cancer mortality in Europe in 2020, and an overview of trends since 1990
Source: Eur J Cancer Prev. 2025 Jun 26;35(3):193–210. doi: 10.1097/CEJ.0000000000000981 (PMC13011951; doi:10.1097/CEJ.0000000000000981)
Supplement: Supplementary file 1 [file ejcp-35-193-s001.docx]

**SUPPLEMENTARY MATERIAL**

**Manuscript title:** Cancer mortality in Europe in 2020, and an overview of trends since 1990

**Table of contents**

[**Table S1.** List of 33 European countries considered, with available calendar years, data quality and population in 2020. 2](#_Toc188956799)

[**Table S2.** Age-standardized mortality rates per 100,000 (first row) and number of deaths (second row) from selected cancer sites among males aged 35-65 years in 33 European countries and the EU-27 in 2020^a^. 3](#_Toc188956800)

[**Table S3.** Age-standardized mortality rates per 100,000 (first row) and number of deaths (second row) from selected cancer sites among females aged 35-64 years in 33 European countries and the EU-27 in 2020^a^. 5](#_Toc188956801)

[**Table S4.** Results of joinpoint analysis for 23 selected cancer sites and all neoplasms in the EU-27 among males and females of all ages and the age group 35-64 years, from 1990 to 2020. 7](#_Toc188956802)

[**Table S5**. Results of joinpoint analysis for mortality from all neoplasms in 23 selected European countries, among males and females of all ages and the 35-64 age group, from 1990 up to the most recent calendar year available. 10](#_Toc188956803)

[**Table S6**. Results of joinpoint analysis for mortality from colorectal cancer in 23 selected European countries, among males and females of all ages and the 35-64 age group, from 1990 up to the most recent calendar year available. 13](#_Toc188956804)

[**Table S7**. Results of joinpoint analysis for mortality from pancreatic cancer in 23 selected European countries, among males and females of all ages and the 35-64 age group, from 1990 up to the most recent calendar year available. 16](#_Toc188956805)

[**Table S8**. Results of joinpoint analysis for mortality from lung cancer in 23 selected European countries, among males and females of all ages and the 35-64 age group, from 1990 up to the most recent calendar year available. 19](#_Toc188956806)

[**Table S9**. Results of joinpoint analysis for mortality from breast cancer in 23 selected European countries among females of all ages and the 35-64 age group, from 1990 up to the most recent calendar year available. 22](#_Toc188956807)

[**Table S10**. Results of joinpoint analysis for mortality from prostate cancer in 23 selected European countries, among males of all ages and the 35-64 age group, from 1990 up to the most recent calendar year available. 24](#_Toc188956808)

[**Figure S1.** Age-standardized mortality rates from colorectal cancer per 100,000 males and females in 33 European countries and the EU-27 in 2020^a^. 26](#_Toc188956809)

[**Figure S2.** Age-standardized mortality rates from pancreatic cancer per 100,000 males and females in 33 European countries and the EU-27 in 2020^a^. 27](#_Toc188956810)

[**Figure S3.** Age-standardized mortality rates from lung cancer per 100,000 males and females in 33 European countries and the EU-27 in 2020^a^. 28](#_Toc188956811)

[**Figure S4.** Age-standardized mortality rates from breast cancer per 100,000 females in 33 European countries and the EU-27 in 2020^a^. 29](#_Toc188956812)

[**Figure S5.** Age-standardized mortality rates from prostate cancer per 100,000 males in 33 European countries and the EU-27 in 2020^a^. 30](#_Toc188956813)

# **Table S1.** List of 33 European countries considered, with available calendar years, data quality and population in 2020.

| **Country** | **Calendar years** | **Data quality^a^** | **Population in 2020^b^** |
| --- | --- | --- | --- |
|  |  |  |  |
| Austria | 1990-2021 | high | > 5 million |
| Belarus | 1990-2018 | high | > 5 million |
| Belgium | 1990-2020 | high | > 10 million |
| Bulgaria | 1990-2021 | medium | > 5 million |
| Croatia | 1990-2021 | high | < 5 million |
| Czech Republic | 1990-2021 | high | > 10 million |
| Denmark | 1990-2021 | high | > 5 million |
| Estonia | 1990-2020 | high | < 5 million |
| Finland | 1990-2021 | high | > 5 million |
| France | 1990-2020 | high | > 50 million |
| Germany | 1990-2020 | high | > 50 million |
| Greece | 1990-2020 | medium | > 10 million |
| Hungary | 1990-2020 | high | > 5 million |
| Iceland | 1990-2021 | high | < 5 million |
| Ireland | 1990-2020 | high | < 5 million |
| Italy | 1990-2020 | high | > 50 million |
| Latvia | 1990-2021 | high | < 5 million |
| Lithuania | 1990-2022 | high | < 5 million |
| Luxembourg | 1990-2022 | high | < 5 million |
| Malta | 1990-2017 | high | < 5 million |
| Netherlands | 1990-2022 | high | > 10 million |
| North Macedonia | 1990-2021 | medium | < 5 million |
| Norway | 1990-2016 | high | > 5 million |
| Poland | 1990-2021 | medium | > 30 million |
| Portugal | 1990-2019 | high | > 10 million |
| Romania | 1990-2019 | high | > 10 million |
| Serbia | 1990-2022 | high | > 5 million |
| Slovakia | 1990-2021 | high | > 5 million |
| Slovenia | 1990-2020 | high | < 5 million |
| Spain | 1990-2021 | high | > 40 million |
| Sweden | 1990-2022 | high | > 10 million |
| Switzerland | 1990-2020 | high | > 5 million |
| United Kingdom | 1990-2020 | high | > 50 million |
|  |  |  |  |

^a^ World Health Organization. WHO assessment as of November 2020 based on most recent years of available country data. Information on the assessment method is published in WHO methods and data sources for country-level causes of death 2000-2019, pp7-15, WHO, Dec 2020.

^b^ For Portugal and Romania: 2019; for Belarus: 2018; for Malta: 2017; and for Norway: 2016.

# **Table S2.** Age-standardized mortality rates per 100,000 (first row) and number of deaths (second row) from selected cancer sites among males aged 35-65 years in 33 European countries and the EU-27 in 2020^a^.

|  | Oral cavity/  pharynx | Esophagus | Stomach | Colorectum | Liver | Gallbladder | Pancreas | Larynx | Lung | Bone | Connective/  soft tissue sarcomas | Skin | Prostate | Testis | Bladder | Kidney | Thyroid | HL | NHL | MM | Leukemias | All neoplasms |
| --- | --- | --- | --- | --- | --- | --- | --- | --- | --- | --- | --- | --- | --- | --- | --- | --- | --- | --- | --- | --- | --- | --- |
| ICD-10 | C00-C14 | C15 | C16 | C17-C21, C26 | C22.0-C22.7 | C23-C24 | C25 | C32 | C33-C34 | C40-C41 | C47, C49 | C43-C44 | C61 | C62 | C67 | C64-C66, C68 | C73 | C81 | C82-C85, C96 | C88, C90 | C91-C95 | C00-D48 |
|  |  |  |  |  |  |  |  |  |  |  |  |  |  |  |  |  |  |  |  |  |  |  |
| Austria | 6.32 | 5.40 | 4.51 | 11.46 | 5.25 | 1.53 | 9.90 | 1.72 | 29.93 | 0.46 | 1.08 | 2.85 | 3.33 | 0.42 | 1.77 | 3.45 | 0.26 | 0.35 | 2.95 | 0.99 | 2.54 | 112.10 |
|  | 142 | 119 | 97 | 251 | 121 | 32 | 224 | 40 | 676 | 9 | 23 | 60 | 77 | 8 | 40 | 78 | 6 | 6 | 64 | 22 | 56 | 2489 |
| Belarus | 29.20 | 11.89 | 23.57 | 21.56 | 6.15 | 1.14 | 14.54 | 9.59 | 64.01 | 0.46 | 1.14 | 3.27 | 7.40 | 0.43 | 4.53 | 9.53 | 0.28 | 1.28 | 5.01 | 1.87 | 5.41 | 248.99 |
|  | 601 | 247 | 496 | 459 | 126 | 24 | 308 | 200 | 1378 | 10 | 22 | 65 | 162 | 8 | 99 | 206 | 6 | 24 | 103 | 39 | 114 | 5252 |
| Belgium | 5.69 | 5.58 | 4.25 | 10.80 | 3.72 | 0.27 | 8.14 | 1.22 | 29.19 | 0.49 | 0.88 | 2.24 | 2.86 | 0.29 | 2.37 | 3.27 | 0.22 | 0.16 | 2.30 | 1.12 | 2.87 | 109.65 |
|  | 156 | 151 | 112 | 287 | 100 | 7 | 221 | 33 | 799 | 13 | 23 | 57 | 81 | 7 | 65 | 86 | 6 | 4 | 61 | 31 | 72 | 2939 |
| Bulgaria | 8.91 | 5.16 | 11.32 | 23.23 | 4.57 | 1.12 | 15.69 | 9.10 | 52.61 | 1.03 | 0.85 | 3.09 | 5.40 | 1.40 | 4.92 | 6.65 | 0.42 | 0.67 | 3.37 | 1.46 | 4.87 | 192.54 |
|  | 143 | 84 | 182 | 382 | 77 | 19 | 258 | 150 | 877 | 17 | 13 | 49 | 91 | 21 | 82 | 109 | 7 | 11 | 53 | 24 | 76 | 3160 |
| Croatia | 14.01 | 5.26 | 10.87 | 25.16 | 5.03 | 1.97 | 9.76 | 4.13 | 55.52 | 1.04 | 0.83 | 5.63 | 3.97 | 1.05 | 4.65 | 6.62 | 0.77 | 0.37 | 4.05 | 1.94 | 3.63 | 186.43 |
|  | 137 | 52 | 107 | 249 | 52 | 19 | 97 | 41 | 576 | 10 | 8 | 51 | 42 | 8 | 47 | 64 | 7 | 3 | 39 | 20 | 36 | 1859 |
| Czech Republic | 11.81 | 6.98 | 6.10 | 17.96 | 3.20 | 2.12 | 13.31 | 2.51 | 27.39 | 0.53 | 0.90 | 3.08 | 4.65 | 0.74 | 3.12 | 6.15 | 0.34 | 0.37 | 2.44 | 2.20 | 3.41 | 140.52 |
|  | 281 | 167 | 145 | 428 | 77 | 51 | 319 | 60 | 660 | 12 | 21 | 73 | 113 | 17 | 75 | 148 | 8 | 9 | 58 | 53 | 82 | 3363 |
| Denmark | 5.28 | 5.14 | 5.53 | 12.04 | 4.04 | 0.74 | 8.90 | 0.68 | 21.67 | 0.08 | 0.86 | 2.74 | 4.30 | 0.41 | 1.63 | 3.57 | 0.37 | 0.00 | 1.79 | 0.89 | 2.50 | 103.43 |
|  | 73 | 70 | 70 | 155 | 52 | 9 | 119 | 9 | 292 | 1 | 11 | 35 | 58 | 4 | 22 | 47 | 5 | 0 | 23 | 12 | 30 | 1360 |
| Estonia | 16.44 | 11.74 | 16.56 | 7.71 | 8.11 | 1.77 | 11.59 | 0.98 | 42.93 | 0.65 | 1.82 | 3.87 | 7.51 | 0.00 | 3.81 | 6.66 | - | 0.00 | 3.33 | 1.37 | 4.18 | 171.86 |
|  | 47 | 34 | 48 | 22 | 24 | 5 | 34 | 3 | 126 | 2 | 5 | 11 | 22 | 0 | 11 | 20 | - | 0 | 9 | 4 | 12 | 498 |
| Finland | 3.72 | 5.31 | 4.14 | 12.00 | 5.51 | 2.14 | 9.16 | 0.38 | 18.77 | 0.07 | 1.01 | 2.68 | 3.43 | 0.42 | 1.59 | 3.20 | 0.92 | 0.22 | 3.40 | 1.46 | 1.62 | 94.95 |
|  | 50 | 68 | 51 | 149 | 70 | 27 | 117 | 5 | 243 | 1 | 11 | 33 | 45 | 4 | 21 | 41 | 12 | 3 | 42 | 19 | 20 | 1198 |
| France | 7.57 | 5.48 | 4.96 | 12.06 | 7.89 | 0.47 | 9.79 | 1.56 | 40.29 | 0.68 | 1.08 | 2.48 | 3.01 | 0.24 | 3.08 | 4.55 | 0.23 | 0.24 | 2.98 | 1.13 | 2.96 | 137.78 |
|  | 1105 | 803 | 707 | 1743 | 1174 | 66 | 1426 | 231 | 5911 | 92 | 147 | 340 | 455 | 28 | 458 | 662 | 32 | 32 | 426 | 164 | 421 | 20007 |
| Germany | 7.39 | 6.88 | 5.92 | 13.56 | 4.88 | 1.36 | 10.12 | 1.35 | 31.28 | 0.44 | 1.12 | 2.38 | 4.18 | 0.67 | 1.88 | 4.63 | 0.35 | 0.22 | 2.93 | 1.33 | 3.14 | 122.77 |
|  | 1610 | 1499 | 1245 | 2892 | 1050 | 296 | 2193 | 303 | 6884 | 83 | 217 | 472 | 935 | 116 | 408 | 1010 | 74 | 45 | 609 | 292 | 650 | 26346 |
| Greece | 3.88 | 3.23 | 7.32 | 11.32 | 3.94 | 0.91 | 11.08 | 3.55 | 47.02 | 0.59 | 1.70 | 1.67 | 2.50 | 0.34 | 4.96 | 4.33 | 0.67 | 0.61 | 3.18 | 1.54 | 4.15 | 145.86 |
|  | 93 | 78 | 176 | 273 | 96 | 22 | 265 | 88 | 1160 | 14 | 39 | 40 | 62 | 7 | 123 | 106 | 16 | 14 | 76 | 38 | 101 | 3550 |
| Hungary | 25.40 | 7.40 | 9.64 | 34.28 | 2.54 | 2.82 | 16.96 | 10.42 | 71.91 | 0.39 | 1.18 | 3.75 | 5.42 | 0.97 | 5.45 | 7.26 | 0.51 | 0.38 | 4.43 | 1.47 | 3.82 | 240.49 |
|  | 548 | 161 | 207 | 743 | 56 | 61 | 365 | 225 | 1572 | 8 | 25 | 80 | 120 | 20 | 120 | 157 | 11 | 8 | 94 | 32 | 82 | 5212 |
| Iceland | 1.24 | 9.46 | 4.15 | 17.74 | 4.13 | 0.00 | 7.53 | 0.00 | 6.07 | 0.00 | 1.18 | 1.24 | 4.89 | - | 0.00 | 3.59 | 0.00 | - | 1.24 | 0.00 | 0.00 | 79.79 |
|  | 1 | 7 | 3 | 14 | 3 | 0 | 6 | 0 | 5 | 0 | 1 | 1 | 4 | - | 0 | 3 | 0 | - | 1 | 0 | 0 | 63 |
| Ireland | 5.29 | 8.69 | 5.03 | 13.37 | 5.11 | 0.00 | 7.62 | 1.17 | 23.24 | 0.61 | 0.78 | 3.80 | 3.95 | 0.29 | 1.33 | 4.23 | 0.00 | 0.19 | 2.15 | 1.76 | 2.68 | 104.30 |
|  | 54 | 89 | 51 | 136 | 52 | 0 | 78 | 12 | 239 | 6 | 8 | 38 | 40 | 3 | 14 | 43 | 0 | 2 | 23 | 18 | 27 | 1066 |
| Italy | 4.43 | 2.64 | 6.07 | 12.53 | 6.12 | 1.59 | 9.20 | 1.76 | 24.97 | 0.46 | 1.16 | 2.99 | 2.17 | 0.42 | 2.26 | 3.96 | 0.31 | 0.44 | 3.16 | 1.61 | 3.45 | 110.19 |
|  | 657 | 388 | 883 | 1836 | 914 | 236 | 1358 | 267 | 3733 | 64 | 156 | 431 | 329 | 50 | 341 | 581 | 45 | 61 | 456 | 240 | 492 | 16180 |
| Latvia | 16.63 | 10.29 | 17.03 | 16.36 | 7.66 | 0.93 | 15.70 | 8.72 | 47.91 | 0.53 | 1.30 | 2.68 | 7.56 | 0.81 | 7.40 | 5.79 | 0.00 | 0.29 | 3.05 | 2.51 | 3.63 | 203.12 |
|  | 68 | 43 | 71 | 71 | 32 | 4 | 67 | 38 | 208 | 2 | 5 | 10 | 34 | 3 | 32 | 24 | 0 | 1 | 13 | 11 | 15 | 860 |
| Lithuania | 28.73 | 14.22 | 19.54 | 17.58 | 8.99 | 0.91 | 13.10 | 6.63 | 43.06 | 1.09 | 1.55 | 5.30 | 7.02 | 0.35 | 5.96 | 7.76 | 0.17 | 0.22 | 3.00 | 2.25 | 5.19 | 217.93 |
|  | 183 | 95 | 124 | 115 | 59 | 6 | 86 | 46 | 287 | 7 | 9 | 35 | 49 | 2 | 41 | 52 | 1 | 1 | 18 | 14 | 33 | 1424 |
| Luxembourg | 2.70 | 5.69 | 8.52 | 9.21 | 4.63 | 0.58 | 5.23 | 0.73 | 22.80 | 0.00 | 0.78 | 2.10 | 4.45 | 0.80 | 2.18 | 4.06 | 0.00 | - | 1.96 | 0.00 | 1.97 | 87.03 |
|  | 4 | 8 | 12 | 13 | 7 | 1 | 8 | 1 | 34 | 0 | 1 | 3 | 6 | 1 | 3 | 6 | 0 | - | 3 | 0 | 3 | 127 |
| Malta | 6.49 | 2.82 | 3.89 | 7.88 | 7.44 | 0.00 | 12.19 | 0.82 | 28.42 | 0.00 | 0.87 | 0.82 | 3.38 | 1.16 | 2.86 | 3.73 | 0.00 | 1.16 | 3.38 | 0.87 | 5.24 | 108.35 |
|  | 7 | 3 | 4 | 9 | 8 | 0 | 13 | 1 | 32 | 0 | 1 | 1 | 4 | 1 | 3 | 4 | 0 | 1 | 4 | 1 | 6 | 119 |
| Netherlands | 3.60 | 8.52 | 3.49 | 15.25 | 2.85 | 1.05 | 7.96 | 0.79 | 26.47 | 0.20 | 1.21 | 3.69 | 3.74 | 0.39 | 2.20 | 4.51 | 0.39 | 0.25 | 3.45 | 1.61 | 2.89 | 112.17 |
|  | 153 | 363 | 144 | 623 | 119 | 44 | 337 | 34 | 1129 | 8 | 47 | 143 | 166 | 12 | 94 | 193 | 16 | 10 | 138 | 66 | 119 | 4669 |
| North Macedonia | 4.25 | 2.21 | 9.46 | 14.95 | 9.16 | 1.10 | 6.71 | 4.24 | 59.40 | 1.45 | 0.39 | 4.79 | 5.22 | 0.62 | 2.42 | 3.59 | 0.60 | 1.39 | 2.52 | 0.90 | 3.81 | 159.74 |
|  | 20 | 11 | 45 | 72 | 44 | 5 | 32 | 21 | 289 | 7 | 2 | 22 | 26 | 3 | 12 | 17 | 3 | 6 | 12 | 4 | 18 | 769 |
| Norway | 3.24 | 3.91 | 4.11 | 14.35 | 5.92 | 1.00 | 6.79 | 0.34 | 20.49 | 0.43 | 0.73 | 4.56 | 4.56 | 0.10 | 1.67 | 3.79 | 0.28 | 0.17 | 2.56 | 2.51 | 2.41 | 95.83 |
|  | 37 | 45 | 48 | 162 | 68 | 11 | 78 | 4 | 237 | 5 | 8 | 50 | 53 | 1 | 19 | 44 | 3 | 2 | 29 | 29 | 27 | 1092 |
| Poland | 13.84 | 6.43 | 10.41 | 20.01 | 2.53 | 1.71 | 9.83 | 6.37 | 45.35 | 0.62 | 1.19 | 3.48 | 5.35 | 0.81 | 5.74 | 5.28 | 0.28 | 0.58 | 3.37 | 1.81 | 3.62 | 179.48 |
|  | 1173 | 558 | 893 | 1741 | 220 | 150 | 846 | 554 | 4074 | 54 | 97 | 293 | 497 | 64 | 523 | 460 | 24 | 48 | 281 | 160 | 312 | 15689 |
| Portugal | 15.50 | 9.60 | 14.89 | 19.77 | 11.29 | 1.22 | 8.91 | 5.18 | 43.21 | 0.66 | 1.59 | 2.53 | 3.92 | 0.65 | 3.22 | 4.28 | 0.50 | 0.28 | 4.21 | 1.77 | 4.42 | 178.60 |
|  | 366 | 227 | 347 | 461 | 270 | 29 | 210 | 122 | 1021 | 15 | 36 | 57 | 95 | 13 | 77 | 101 | 12 | 6 | 95 | 42 | 101 | 4190 |
| Romania | 24.65 | 7.31 | 14.86 | 22.71 | 5.12 | 1.51 | 13.08 | 10.03 | 67.83 | 1.75 | 0.88 | 3.66 | 4.16 | 0.70 | 5.20 | 5.55 | 0.44 | 0.42 | 3.28 | 1.43 | 3.91 | 234.52 |
|  | 1083 | 323 | 656 | 1008 | 228 | 68 | 581 | 447 | 3034 | 76 | 37 | 160 | 191 | 29 | 235 | 243 | 19 | 18 | 143 | 62 | 171 | 10401 |
| Serbia | 11.55 | 4.33 | 9.13 | 23.09 | 7.22 | 1.34 | 12.06 | 5.49 | 69.99 | 0.96 | 1.33 | 3.57 | 5.41 | 1.29 | 6.06 | 5.47 | 0.74 | 0.93 | 4.79 | 2.35 | 5.19 | 208.77 |
|  | 185 | 71 | 149 | 367 | 119 | 22 | 196 | 90 | 1161 | 15 | 20 | 54 | 94 | 17 | 101 | 87 | 12 | 13 | 74 | 37 | 81 | 3386 |
| Slovakia | 21.67 | 8.49 | 9.02 | 25.38 | 3.65 | 1.98 | 14.67 | 4.56 | 34.38 | 1.07 | 1.95 | 2.96 | 5.77 | 1.46 | 3.34 | 7.51 | 0.22 | 0.47 | 4.09 | 2.07 | 3.86 | 175.76 |
|  | 271 | 107 | 111 | 318 | 46 | 25 | 183 | 59 | 434 | 13 | 23 | 36 | 74 | 17 | 43 | 95 | 3 | 6 | 51 | 26 | 49 | 2203 |
| Slovenia | 12.03 | 7.12 | 7.39 | 13.23 | 10.11 | 1.80 | 8.91 | 2.68 | 35.67 | 1.04 | 0.87 | 3.24 | 6.37 | 0.73 | 4.38 | 3.16 | 0.39 | 0.77 | 2.84 | 1.74 | 2.49 | 139.43 |
|  | 64 | 37 | 39 | 70 | 56 | 10 | 46 | 14 | 192 | 5 | 4 | 17 | 35 | 3 | 24 | 17 | 2 | 4 | 15 | 9 | 13 | 742 |
| Spain | 5.86 | 4.36 | 6.12 | 14.79 | 7.99 | 0.79 | 9.39 | 2.76 | 37.73 | 0.43 | 1.26 | 1.93 | 2.68 | 0.22 | 3.48 | 4.79 | 0.25 | 0.35 | 2.96 | 1.31 | 2.52 | 128.90 |
|  | 680 | 505 | 698 | 1697 | 923 | 90 | 1081 | 322 | 4364 | 46 | 139 | 217 | 310 | 22 | 399 | 552 | 28 | 39 | 335 | 152 | 279 | 14802 |
| Sweden | 2.11 | 3.01 | 3.03 | 11.87 | 3.13 | 1.10 | 8.65 | 0.37 | 10.12 | 0.58 | 0.96 | 3.89 | 3.64 | 0.15 | 1.08 | 2.83 | 0.23 | 0.09 | 1.88 | 1.90 | 2.11 | 75.51 |
|  | 46 | 65 | 64 | 256 | 68 | 24 | 192 | 8 | 226 | 11 | 20 | 83 | 82 | 3 | 24 | 62 | 5 | 2 | 41 | 42 | 45 | 1643 |
| Switzerland | 4.66 | 4.49 | 3.78 | 9.35 | 4.43 | 1.08 | 7.69 | 0.71 | 21.46 | 0.14 | 1.16 | 1.75 | 2.85 | 0.46 | 1.88 | 2.29 | 0.33 | 0.19 | 1.80 | 1.32 | 1.79 | 84.13 |
|  | 97 | 94 | 75 | 190 | 91 | 22 | 159 | 14 | 448 | 3 | 21 | 35 | 59 | 9 | 39 | 46 | 7 | 4 | 37 | 28 | 36 | 1729 |
| United Kingdom | 5.50 | 9.01 | 3.78 | 16.38 | 5.17 | 0.57 | 7.64 | 0.96 | 21.76 | 0.34 | 1.23 | 2.74 | 4.15 | 0.22 | 2.00 | 4.39 | 0.20 | 0.22 | 3.21 | 1.52 | 2.48 | 110.72 |
|  | 804 | 1321 | 535 | 2328 | 750 | 83 | 1116 | 143 | 3185 | 47 | 171 | 384 | 628 | 29 | 292 | 633 | 29 | 31 | 456 | 224 | 348 | 15982 |
| EU-27 | 8.70 | 5.74 | 6.97 | 15.16 | 5.57 | 1.23 | 10.14 | 2.91 | 36.11 | 0.57 | 1.14 | 2.79 | 3.67 | 0.52 | 3.08 | 4.69 | 0.32 | 0.34 | 3.07 | 1.46 | 3.21 | 138.44 |
|  | 9191 | 6103 | 7243 | 15929 | 5951 | 1303 | 10726 | 3114 | 38766 | 569 | 1125 | 2825 | 4011 | 464 | 3324 | 4962 | 339 | 334 | 3168 | 1554 | 3298 | 146080 |

^a^ Available year for Portugal and Romania: 2019; for Belarus: 2018; for Malta: 2017; for Norway: 2016.

International Classification of Diseases 10th Revision (ICD-10).

# **Table S3.** Age-standardized mortality rates per 100,000 (first row) and number of deaths (second row) from selected cancer sites among females aged 35-64 years in 33 European countries and the EU-27 in 2020^a^.

|  | Oral cavity/  pharynx | Esophagus | Stomach | Colorectum | Liver | Gallbladder | Pancreas | Larynx | Lung | Bone | Connecti  ve/soft tissue sarcomas | Skin | Breast | Uterus | Ovary | Bladder | Kidney | Thyroid | HL | NHL | MM | Leukemias | All neoplasms |
| --- | --- | --- | --- | --- | --- | --- | --- | --- | --- | --- | --- | --- | --- | --- | --- | --- | --- | --- | --- | --- | --- | --- | --- |
| IC-D10 | C00-C14 | C15 | C16 | C17-C21, C26 | C22.0-C22.7 | C23-C24 | C25 | C32 | C33-C34 | C40-C41 | C47, C49 | C43-C44 | C50 | C53-C55 | C56-C57.4 | C67 | C64-C66, C68 | C73 | C81 | C82-C85, C96 | C88, C90 | C91-C95 | C00-D48 |
|  |  |  |  |  |  |  |  |  |  |  |  |  |  |  |  |  |  |  |  |  |  |  |  |
| Austria | 1.53 | 1.14 | 2.91 | 7.72 | 1.79 | 0.89 | 7.18 | 0.19 | 18.30 | 0.30 | 0.92 | 1.83 | 17.98 | 5.16 | 6.16 | 0.86 | 0.78 | 0.16 | 0.14 | 1.47 | 0.90 | 1.68 | 90.03 |
|  | 36 | 25 | 65 | 166 | 39 | 20 | 166 | 4 | 425 | 7 | 21 | 39 | 374 | 114 | 135 | 20 | 18 | 4 | 3 | 32 | 21 | 36 | 1985 |
| Belarus | 2.16 | 0.63 | 8.89 | 13.39 | 1.02 | 1.18 | 4.60 | 0.42 | 5.18 | 0.34 | 0.77 | 2.41 | 22.42 | 13.83 | 9.64 | 0.22 | 1.59 | 0.35 | 0.65 | 2.37 | 1.61 | 3.41 | 112.69 |
|  | 51 | 15 | 218 | 346 | 25 | 29 | 116 | 10 | 132 | 7 | 19 | 60 | 534 | 325 | 237 | 5 | 43 | 8 | 14 | 58 | 41 | 85 | 2766 |
| Belgium | 0.90 | 1.35 | 1.67 | 7.20 | 1.59 | 0.59 | 5.65 | 0.26 | 20.29 | 0.18 | 0.92 | 1.61 | 20.82 | 5.36 | 4.79 | 0.68 | 1.70 | 0.25 | 0.00 | 0.83 | 1.00 | 1.67 | 92.08 |
|  | 24 | 37 | 43 | 192 | 43 | 15 | 150 | 7 | 560 | 4 | 24 | 41 | 526 | 136 | 128 | 18 | 45 | 7 | 0 | 22 | 27 | 45 | 2428 |
| Bulgaria | 1.84 | 0.77 | 5.63 | 13.25 | 2.04 | 0.83 | 7.86 | 0.64 | 19.90 | 0.37 | 0.31 | 1.82 | 25.95 | 19.42 | 10.51 | 1.67 | 2.37 | 0.29 | 0.38 | 1.74 | 1.12 | 2.68 | 138.45 |
|  | 31 | 13 | 91 | 222 | 34 | 14 | 134 | 11 | 339 | 7 | 5 | 30 | 426 | 310 | 172 | 29 | 39 | 5 | 5 | 28 | 20 | 43 | 2291 |
| Croatia | 2.12 | 1.22 | 5.30 | 14.26 | 1.55 | 1.85 | 6.17 | 0.20 | 25.00 | 0.58 | 0.75 | 3.06 | 20.95 | 9.93 | 11.26 | 1.79 | 2.28 | 0.08 | 0.58 | 2.03 | 1.02 | 2.16 | 128.98 |
|  | 22 | 13 | 53 | 144 | 17 | 19 | 63 | 2 | 270 | 5 | 7 | 29 | 202 | 98 | 113 | 17 | 24 | 1 | 5 | 21 | 11 | 22 | 1307 |
| Czech Republic | 2.77 | 1.08 | 3.98 | 11.56 | 1.19 | 2.02 | 7.08 | 0.24 | 14.25 | 0.34 | 0.85 | 1.73 | 18.99 | 8.87 | 8.66 | 1.01 | 2.43 | 0.20 | 0.45 | 1.54 | 1.50 | 2.29 | 107.37 |
|  | 65 | 26 | 94 | 275 | 28 | 50 | 174 | 6 | 346 | 8 | 19 | 41 | 436 | 208 | 206 | 25 | 58 | 5 | 11 | 37 | 36 | 55 | 2550 |
| Denmark | 2.06 | 1.45 | 2.37 | 9.42 | 2.29 | 1.39 | 7.61 | 0.15 | 22.87 | 0.17 | 1.51 | 2.39 | 19.32 | 5.32 | 5.63 | 1.44 | 1.00 | 0.35 | 0.08 | 0.77 | 0.43 | 1.61 | 106.34 |
|  | 28 | 19 | 31 | 123 | 31 | 18 | 100 | 2 | 311 | 2 | 19 | 31 | 237 | 62 | 71 | 19 | 14 | 4 | 1 | 10 | 6 | 21 | 1376 |
| Estonia | 2.45 | 0.92 | 7.00 | 6.93 | 2.68 | 2.04 | 6.02 | 0.00 | 7.82 | 0.00 | 1.37 | 2.43 | 24.22 | 13.39 | 11.09 | 0.55 | 2.30 | 0.70 | 0.00 | 2.08 | 1.82 | 2.13 | 110.87 |
|  | 8 | 3 | 21 | 23 | 9 | 7 | 20 | 0 | 26 | 0 | 4 | 7 | 73 | 43 | 33 | 2 | 8 | 2 | 0 | 7 | 6 | 7 | 351 |
| Finland | 0.87 | 1.50 | 2.80 | 7.93 | 1.85 | 1.48 | 6.02 | 0.14 | 10.30 | 0.19 | 0.94 | 1.69 | 19.68 | 3.62 | 4.74 | 0.66 | 1.69 | 0.47 | 0.11 | 2.16 | 0.61 | 0.51 | 78.08 |
|  | 12 | 19 | 35 | 103 | 24 | 20 | 77 | 2 | 137 | 2 | 11 | 20 | 226 | 43 | 58 | 9 | 23 | 6 | 1 | 27 | 8 | 7 | 968 |
| France | 1.68 | 1.14 | 2.23 | 8.97 | 2.03 | 0.39 | 6.54 | 0.20 | 20.66 | 0.30 | 0.87 | 1.57 | 24.25 | 5.48 | 4.61 | 0.77 | 1.36 | 0.14 | 0.08 | 1.40 | 0.77 | 2.01 | 100.45 |
|  | 260 | 178 | 330 | 1352 | 315 | 63 | 1013 | 33 | 3215 | 40 | 129 | 225 | 3511 | 797 | 712 | 122 | 210 | 22 | 12 | 209 | 120 | 297 | 15128 |
| Germany | 1.64 | 1.48 | 3.28 | 8.73 | 2.16 | 1.21 | 6.67 | 0.22 | 20.30 | 0.24 | 0.97 | 1.60 | 23.16 | 6.40 | 6.39 | 1.00 | 1.88 | 0.20 | 0.11 | 1.62 | 0.80 | 1.98 | 103.63 |
|  | 352 | 324 | 645 | 1829 | 463 | 264 | 1457 | 50 | 4485 | 45 | 195 | 318 | 4619 | 1285 | 1351 | 211 | 411 | 44 | 21 | 334 | 172 | 403 | 21662 |
| Greece | 1.49 | 0.36 | 3.30 | 7.82 | 1.77 | 0.46 | 5.23 | 0.37 | 16.23 | 0.42 | 1.23 | 1.66 | 21.38 | 6.99 | 7.62 | 0.91 | 1.09 | 0.22 | 0.23 | 1.96 | 0.98 | 2.74 | 101.59 |
|  | 38 | 9 | 87 | 206 | 45 | 12 | 141 | 10 | 437 | 11 | 32 | 39 | 545 | 180 | 196 | 24 | 29 | 6 | 6 | 51 | 27 | 71 | 2648 |
| Hungary | 4.50 | 1.21 | 5.50 | 17.78 | 0.75 | 2.64 | 9.30 | 1.18 | 40.42 | 0.23 | 1.25 | 2.85 | 26.54 | 14.28 | 8.90 | 2.31 | 3.04 | 0.48 | 0.16 | 2.45 | 1.53 | 3.80 | 164.04 |
|  | 109 | 31 | 129 | 425 | 18 | 63 | 218 | 29 | 996 | 5 | 28 | 65 | 602 | 324 | 203 | 57 | 72 | 11 | 4 | 58 | 36 | 88 | 3871 |
| Iceland | 0.00 | 1.17 | 1.67 | 12.61 | 0.00 | 4.17 | 3.61 | 0.00 | 21.46 | . | 1.26 | 3.01 | 23.82 | 8.61 | 3.69 | 2.52 | 1.26 | 0.00 | - | 0.00 | 0.00 | 0.00 | 99.35 |
|  | 0 | 1 | 1 | 9 | 0 | 3 | 3 | 0 | 17 | . | 1 | 2 | 17 | 6 | 3 | 2 | 1 | 0 | - | 0 | 0 | 0 | 74 |
| Ireland | 1.06 | 2.83 | 2.94 | 9.64 | 2.85 | 0.66 | 5.13 | 0.30 | 18.22 | 0.49 | 0.83 | 1.74 | 26.45 | 7.61 | 6.79 | 0.88 | 1.74 | 0.09 | 0.10 | 1.34 | 0.85 | 1.07 | 103.39 |
|  | 11 | 30 | 30 | 102 | 30 | 7 | 54 | 3 | 192 | 5 | 9 | 18 | 273 | 79 | 71 | 9 | 18 | 1 | 1 | 14 | 9 | 11 | 1078 |
| Italy | 1.35 | 0.59 | 3.63 | 9.15 | 1.53 | 1.22 | 6.52 | 0.24 | 14.66 | 0.27 | 0.73 | 1.79 | 24.30 | 5.88 | 6.24 | 0.81 | 1.34 | 0.25 | 0.25 | 1.87 | 1.12 | 2.18 | 97.70 |
|  | 208 | 94 | 533 | 1400 | 238 | 189 | 1024 | 38 | 2305 | 38 | 107 | 258 | 3558 | 865 | 950 | 125 | 203 | 39 | 34 | 285 | 179 | 322 | 14758 |
| Latvia | 3.50 | 1.59 | 6.38 | 10.79 | 1.57 | 1.00 | 6.56 | 0.66 | 10.51 | 0.59 | 0.59 | 1.92 | 31.29 | 15.84 | 14.99 | 0.87 | 3.09 | 0.81 | 0.17 | 2.16 | 3.14 | 4.14 | 140.93 |
|  | 19 | 8 | 31 | 50 | 8 | 5 | 34 | 3 | 53 | 3 | 3 | 10 | 142 | 74 | 71 | 5 | 16 | 4 | 1 | 11 | 16 | 20 | 674 |
| Lithuania | 3.01 | 1.84 | 8.36 | 8.88 | 1.87 | 2.15 | 5.42 | 0.15 | 9.23 | 0.27 | 0.87 | 2.96 | 26.13 | 18.89 | 14.22 | 0.96 | 2.18 | 0.27 | 0.00 | 1.26 | 1.21 | 1.56 | 127.64 |
|  | 23 | 13 | 59 | 68 | 15 | 15 | 44 | 1 | 70 | 2 | 7 | 20 | 178 | 130 | 103 | 7 | 15 | 2 | 0 | 10 | 10 | 12 | 921 |
| Luxembourg | 2.29 | 0.00 | 0.79 | 6.03 | 2.19 | 0.00 | 7.32 | 0.00 | 13.12 | . | 0.62 | 0.62 | 13.16 | 1.57 | 2.82 | 0.62 | 0.00 | 0.00 | 0.00 | 0.75 | 0.00 | 1.37 | 60.29 |
|  | 3 | 0 | 1 | 8 | 3 | 0 | 10 | 0 | 19 | . | 1 | 1 | 18 | 2 | 4 | 1 | 0 | 0 | 0 | 1 | 0 | 2 | 83 |
| Malta | 3.59 | 0.87 | 1.74 | 16.67 | 1.44 | 0.00 | 5.24 | 0.00 | 18.71 | 2.12 | 0.83 | 1.74 | 22.10 | 4.04 | 12.76 | 1.74 | 0.83 | - | 0.00 | 3.59 | 0.00 | 1.15 | 109.73 |
|  | 3 | 1 | 2 | 17 | 1 | 0 | 5 | 0 | 21 | 2 | 1 | 2 | 21 | 4 | 12 | 2 | 1 | - | 0 | 3 | 0 | 1 | 109 |
| Netherlands | 1.29 | 2.03 | 2.67 | 11.88 | 2.01 | 0.94 | 6.25 | 0.20 | 27.43 | 0.29 | 0.81 | 2.55 | 24.63 | 5.25 | 5.78 | 0.97 | 1.62 | 0.15 | 0.15 | 1.55 | 0.91 | 1.78 | 112.32 |
|  | 54 | 90 | 105 | 484 | 81 | 39 | 270 | 9 | 1173 | 12 | 31 | 101 | 955 | 201 | 241 | 39 | 66 | 7 | 5 | 65 | 41 | 69 | 4588 |
| North Macedonia | 0.76 | 0.45 | 4.82 | 11.41 | 4.08 | 0.39 | 5.73 | 0.69 | 13.81 | 0.59 | 0.00 | 3.53 | 30.24 | 12.17 | 6.18 | 1.46 | 1.28 | 0.19 | 0.81 | 1.45 | 0.82 | 2.42 | 119.39 |
|  | 4 | 2 | 23 | 55 | 19 | 2 | 27 | 3 | 67 | 3 | 0 | 16 | 144 | 55 | 29 | 7 | 6 | 1 | 4 | 7 | 4 | 11 | 565 |
| Norway | 0.98 | 0.81 | 2.02 | 13.35 | 2.24 | 0.71 | 4.42 | 0.26 | 17.41 | 0.11 | 0.68 | 2.93 | 18.73 | 6.23 | 8.21 | 0.76 | 1.03 | 0.30 | 0.00 | 1.63 | 0.87 | 2.37 | 97.17 |
|  | 11 | 9 | 22 | 146 | 25 | 8 | 49 | 3 | 197 | 1 | 7 | 31 | 201 | 66 | 91 | 9 | 11 | 3 | 0 | 18 | 10 | 26 | 1063 |
| Poland | 2.91 | 1.16 | 4.55 | 12.22 | 1.07 | 2.13 | 6.34 | 0.87 | 21.88 | 0.48 | 0.80 | 1.70 | 24.88 | 11.43 | 11.09 | 1.17 | 1.95 | 0.39 | 0.26 | 1.84 | 1.11 | 2.56 | 131.53 |
|  | 263 | 103 | 406 | 1115 | 101 | 200 | 587 | 79 | 2135 | 43 | 69 | 152 | 2171 | 1016 | 989 | 113 | 183 | 36 | 24 | 169 | 105 | 226 | 12020 |
| Portugal | 1.46 | 0.59 | 6.34 | 11.99 | 2.13 | 1.23 | 4.26 | 0.27 | 13.49 | 0.44 | 1.36 | 1.45 | 24.67 | 8.27 | 3.89 | 0.73 | 1.47 | 0.26 | 0.20 | 2.20 | 1.42 | 2.84 | 102.49 |
|  | 38 | 15 | 162 | 308 | 57 | 34 | 115 | 8 | 352 | 11 | 34 | 36 | 611 | 209 | 103 | 19 | 38 | 7 | 5 | 57 | 37 | 72 | 2630 |
| Romania | 2.51 | 0.90 | 5.47 | 11.85 | 1.65 | 0.92 | 6.16 | 0.57 | 18.59 | 0.89 | 0.36 | 1.97 | 26.26 | 22.95 | 8.87 | 1.28 | 1.61 | 0.54 | 0.26 | 2.08 | 0.95 | 2.91 | 136.89 |
|  | 113 | 41 | 247 | 550 | 77 | 43 | 289 | 26 | 867 | 42 | 16 | 88 | 1174 | 1021 | 398 | 60 | 75 | 26 | 12 | 90 | 46 | 132 | 6223 |
| Serbia | 2.59 | 0.72 | 4.80 | 14.85 | 3.87 | 1.26 | 7.08 | 0.48 | 33.10 | 0.95 | 1.48 | 2.67 | 33.98 | 20.78 | 8.33 | 1.82 | 2.10 | 0.39 | 0.15 | 2.62 | 1.63 | 4.30 | 168.16 |
|  | 44 | 12 | 78 | 253 | 68 | 24 | 127 | 8 | 584 | 18 | 23 | 42 | 560 | 337 | 139 | 32 | 37 | 7 | 3 | 45 | 30 | 69 | 2849 |
| Slovakia | 2.98 | 0.85 | 5.20 | 14.31 | 1.16 | 1.92 | 8.31 | 0.44 | 13.94 | 0.33 | 0.87 | 2.81 | 22.60 | 11.87 | 9.40 | 1.07 | 2.17 | 0.46 | 0.26 | 3.13 | 1.62 | 3.18 | 121.41 |
|  | 39 | 11 | 64 | 184 | 16 | 26 | 110 | 6 | 185 | 4 | 11 | 36 | 286 | 148 | 120 | 15 | 29 | 6 | 3 | 41 | 21 | 40 | 1561 |
| Slovenia | 1.16 | 0.75 | 4.19 | 10.01 | 2.42 | 0.71 | 7.92 | 0.17 | 20.83 | 0.39 | 0.26 | 4.77 | 22.44 | 5.71 | 5.65 | 1.22 | 1.60 | 0.18 | 0.17 | 2.24 | 0.36 | 1.41 | 106.50 |
|  | 6 | 4 | 21 | 47 | 12 | 4 | 39 | 1 | 111 | 2 | 1 | 22 | 108 | 28 | 28 | 6 | 7 | 1 | 1 | 12 | 2 | 7 | 530 |
| Spain | 1.56 | 0.81 | 3.45 | 9.24 | 1.94 | 0.63 | 5.61 | 0.26 | 16.45 | 0.32 | 1.09 | 1.12 | 19.38 | 5.76 | 6.04 | 0.65 | 1.49 | 0.33 | 0.27 | 1.56 | 0.72 | 1.75 | 90.80 |
|  | 186 | 96 | 397 | 1088 | 230 | 74 | 666 | 30 | 1972 | 36 | 124 | 126 | 2211 | 660 | 703 | 78 | 174 | 39 | 30 | 179 | 85 | 197 | 10585 |
| Sweden | 0.73 | 1.30 | 1.93 | 9.33 | 1.21 | 1.86 | 6.15 | 0.15 | 10.24 | 0.06 | 1.24 | 2.67 | 16.80 | 4.65 | 5.44 | 0.47 | 1.16 | 0.25 | 0.13 | 1.04 | 1.02 | 1.52 | 78.67 |
|  | 16 | 28 | 39 | 195 | 25 | 39 | 133 | 3 | 223 | 1 | 25 | 53 | 342 | 95 | 115 | 10 | 25 | 5 | 3 | 23 | 22 | 31 | 1644 |
| Switzerland | 1.51 | 0.68 | 2.74 | 7.00 | 2.12 | 0.82 | 5.73 | 0.00 | 12.73 | 0.34 | 0.81 | 1.42 | 18.36 | 3.31 | 5.19 | 1.46 | 1.04 | 0.08 | 0.05 | 1.08 | 0.65 | 1.77 | 77.16 |
|  | 31 | 14 | 53 | 139 | 45 | 17 | 116 | 0 | 264 | 7 | 15 | 26 | 356 | 64 | 108 | 30 | 21 | 2 | 1 | 22 | 13 | 35 | 1544 |
| United Kingdom | 1.93 | 2.39 | 1.98 | 11.77 | 2.67 | 0.80 | 6.06 | 0.24 | 18.65 | 0.19 | 1.01 | 1.85 | 24.11 | 6.45 | 6.95 | 1.29 | 1.96 | 0.25 | 0.06 | 1.65 | 0.91 | 1.78 | 107.66 |
|  | 289 | 364 | 280 | 1714 | 397 | 119 | 910 | 36 | 2845 | 25 | 142 | 264 | 3434 | 915 | 1037 | 192 | 291 | 40 | 9 | 242 | 139 | 256 | 15798 |
| EU-27 | 1.79 | 1.11 | 3.55 | 9.91 | 1.79 | 1.12 | 6.41 | 0.33 | 19.00 | 0.33 | 0.90 | 1.77 | 23.11 | 7.83 | 6.80 | 0.95 | 1.65 | 0.26 | 0.19 | 1.69 | 0.96 | 2.14 | 106.48 |
|  | 1965 | 1230 | 3720 | 10671 | 1960 | 1240 | 7095 | 363 | 21216 | 337 | 932 | 1808 | 23830 | 8134 | 7284 | 1042 | 1803 | 290 | 189 | 1795 | 1063 | 2239 | 113960 |

^a^ Available year for Portugal and Romania: 2019; for Belarus: 2018; for Malta: 2017; for Norway: 2016.

International Classification of Diseases 10th Revision (ICD-10).

# **Table S4.** Results of joinpoint analysis for 23 selected cancer sites and all neoplasms in the EU-27 among males and females of all ages and the age group 35-64 years, from 1990 to 2020.

| **Sex, age group, cancer site** | **Trend 1** | | **Trend 2** | | **Trend 3** | | **Trend 4** | | **Trend 5** | | **Trend 6** | | **Entire period** |
| --- | --- | --- | --- | --- | --- | --- | --- | --- | --- | --- | --- | --- | --- |
|  | **Years** | **APC** | **Years** | **APC** | **Years** | **APC** | **Years** | **APC** | **Years** | **APC** | **Years** | **APC** | **AAPC** |
|  |  |  |  |  |  |  |  |  |  |  |  |  |  |
| **Males, all ages** |  |  |  |  |  |  |  |  |  |  |  |  |  |
| Oral cavity and pharynx | 1990-1992 | 1.6 | 1992-2002 | -1.2* | 2002-2005 | -2.7 | 2005-2018 | -1.2* | 2018-2020 | -4.2* |  |  | -1.4* |
| Esophagus | 1990-1996 | -0.6* | 1996-2008 | -1.5* | 2008-2020 | -1.1* |  |  |  |  |  |  | -1.2* |
| Stomach | 1990-1994 | -3.1* | 1994-1997 | -4* | 1997-2008 | -3.5* | 2008-2017 | -2.9* | 2017-2020 | -3.7* |  |  | -3.3* |
| Colorectum | 1990-1998 | 0.1 | 1998-2012 | -1.1* | 2012-2020 | -1.7* |  |  |  |  |  |  | -1* |
| Liver | 1990-1994 | 5.4* | 1994-2004 | -2.6* | 2004-2012 | 1.9* | 2012-2020 | -0.6 |  |  |  |  | 0.2 |
| Gallbladder and bile ducts | 1990-1997 | -0.9* | 1997-2009 | -2.2* | 2009-2015 | 0.3 | 2015-2020 | -1.5* |  |  |  |  | -1.3* |
| Pancreas | 1990-2002 | 0.1 | 2002-2008 | 0.7* | 2008-2020 | 0.1* |  |  |  |  |  |  | 0.2* |
| Larynx | 1990-1997 | -2.7* | 1997-2001 | -4.4* | 2001-2005 | -2.4* | 2005-2012 | -3.8* | 2012-2017 | -2.5* | 2017-2020 | -4.3* | -3.3* |
| Lung | 1990-1994 | -0.2 | 1994-2008 | -1.6* | 2008-2016 | -2* | 2016-2020 | -3.9* |  |  |  |  | -1.9* |
| Bone | 1990-2004 | -3* | 2004-2020 | -2.2* |  |  |  |  |  |  |  |  | -2.6* |
| Connective and soft tissue | 1990-2004 | -0.2 | 2004-2020 | 1.3* |  |  |  |  |  |  |  |  | 0.6* |
| Skin, incl. melanoma | 1990-2015 | 0.5* | 2015-2018 | -3.2 | 2018-2020 | 2.3 |  |  |  |  |  |  | 0.3 |
| Prostate | 1990-1996 | 0.5* | 1996-2003 | -1.4* | 2003-2006 | -3.1* | 2006-2013 | -1.9* | 2013-2020 | -1.1* |  |  | -1.2* |
| Testis | 1990-1998 | -4.2* | 1998-2020 | -1* |  |  |  |  |  |  |  |  | -1.9* |
| Bladder | 1990-1992 | 0.6 | 1992-2008 | -1.7* | 2008-2011 | -0.1 | 2011-2020 | -1.9* |  |  |  |  | -1.5* |
| Kidney | 1990-1994 | 0.4 | 1994-2006 | -0.9* | 2006-2015 | 0.9* | 2015-2020 | -1.1* |  |  |  |  | -0.2* |
| Thyroid | 1990-2000 | -1.2* | 2000-2005 | -4.3* | 2005-2011 | 0.6 | 2011-2020 | -1.9* |  |  |  |  | -1.6* |
| HL | 1990-2001 | -5.2* | 2001-2020 | -3.6* |  |  |  |  |  |  |  |  | -4.2* |
| NHL | 1990-1996 | 1.4* | 1996-2001 | -0.4 | 2001-2005 | -3.2* | 2005-2011 | -1* | 2011-2014 | 0.1 | 2014-2020 | -1.4* | -0.7* |
| MM | 1990-2000 | 1.4* | 2000-2020 | -1.1* |  |  |  |  |  |  |  |  | -0.3* |
| Leukemias | 1990-2002 | -0.8* | 2002-2020 | -1.5* |  |  |  |  |  |  |  |  | -1.2* |
| All neoplasms | 1990-1993 | 0 | 1993-2000 | -1* | 2000-2018 | -1.5* | 2018-2020 | -2.8* |  |  |  |  | -1.3* |
| **Males, 35-64 years** |  |  |  |  |  |  |  |  |  |  |  |  |  |
| Oral cavity and pharynx | 1990-1998 | -0.4 | 1998-2014 | -2* | 2014-2020 | -3.8* |  |  |  |  |  |  | -2* |
| Esophagus | 1990-2002 | -1.3* | 2002-2020 | -2.3* |  |  |  |  |  |  |  |  | -1.9* |
| Stomach | 1990-2003 | -3.7* | 2003-2017 | -2.8* | 2017-2020 | -4.3* |  |  |  |  |  |  | -3.3* |
| Colorectum | 1990-1998 | -0.3 | 1998-2018 | -1.4* | 2018-2020 | -3.7* |  |  |  |  |  |  | -1.2* |
| Liver | 1990-1994 | 4.6* | 1994-2003 | -3.4* | 2003-2013 | 1.7* | 2013-2020 | -1.6* |  |  |  |  | -0.3 |
| Gallbladder and bile ducts | 1990-2009 | -2* | 2009-2020 | -0.8* |  |  |  |  |  |  |  |  | -1.6* |
| Pancreas | 1990-2007 | 0.1* | 2007-2020 | -0.7* |  |  |  |  |  |  |  |  | -0.2* |
| Larynx | 1990-1994 | -2.2* | 1994-2001 | -4.2* | 2001-2005 | -2.3* | 2005-2020 | -4.7* |  |  |  |  | -3.9* |
| Lung | 1990-1993 | -0.8* | 1993-2002 | -2.2* | 2002-2006 | -1.5* | 2006-2013 | -2.6* | 2013-2018 | -4.4* | 2018-2020 | -6.9* | -2.7* |
| Bone | 1990-2020 | -3.4* |  |  |  |  |  |  |  |  |  |  | -3.4* |
| Connective and soft tissue | 1990-2003 | -0.6* | 2003-2020 | 1* |  |  |  |  |  |  |  |  | 0.3* |
| Skin, incl. melanoma | 1990-2011 | -0.1 | 2011-2020 | -2.5* |  |  |  |  |  |  |  |  | -0.8* |
| Prostate | 1990-2001 | -0.5* | 2001-2018 | -1.8* | 2018-2020 | -4.6 |  |  |  |  |  |  | -1.5* |
| Testis | 1990-2002 | -2.2* | 2002-2020 | 0.5 |  |  |  |  |  |  |  |  | -0.6* |
| Bladder | 1990-2001 | -2.4* | 2001-2012 | -1.2* | 2012-2020 | -3.9* |  |  |  |  |  |  | -2.4* |
| Kidney | 1990-2007 | -1.3* | 2007-2015 | -0.3 | 2015-2020 | -3* |  |  |  |  |  |  | -1.3* |
| Thyroid | 1990-2020 | -2.2* |  |  |  |  |  |  |  |  |  |  | -2.2* |
| HL | 1990-2003 | -6.2* | 2003-2020 | -3.8* |  |  |  |  |  |  |  |  | -4.9* |
| NHL | 1990-1999 | 0.7* | 1999-2006 | -3.8* | 2006-2020 | -2.1* |  |  |  |  |  |  | -1.7* |
| MM | 1990-2000 | 0.5 | 2000-2020 | -2* |  |  |  |  |  |  |  |  | -1.2* |
| Leukemias | 1990-1999 | -1.1* | 1999-2020 | -2.4* |  |  |  |  |  |  |  |  | -2* |
| All neoplasms | 1990-1992 | 0 | 1992-2004 | -1.6* | 2004-2013 | -2* | 2013-2018 | -2.7* | 2018-2020 | -4.3* |  |  | -2* |
| **Females, all ages** |  |  |  |  |  |  |  |  |  |  |  |  |  |
| Oral cavity and pharynx | 1990-1998 | 1.3* | 1998-2008 | 0 | 2008-2018 | 0.8* | 2018-2020 | -3.9 |  |  |  |  | 0.4* |
| Esophagus | 1990-2020 | -0.1* |  |  |  |  |  |  |  |  |  |  | -0.1* |
| Stomach | 1990-2006 | -3.6* | 2006-2020 | -2.8* |  |  |  |  |  |  |  |  | -3.2* |
| Colorectum | 1990-1993 | 0.1 | 1993-2020 | -1.6* |  |  |  |  |  |  |  |  | -1.4* |
| Liver | 1990-1995 | 2.7* | 1995-2000 | -6.4* | 2000-2007 | -0.2 | 2007-2011 | 2.8 | 2011-2020 | 0.3 |  |  | -0.2 |
| Gallbladder and bile ducts | 1990-1997 | -2.4* | 1997-2008 | -3.7* | 2008-2020 | -2.5* |  |  |  |  |  |  | -2.9* |
| Pancreas | 1990-1993 | 1.7* | 1993-1996 | -0.5 | 1996-2001 | 1.1* | 2001-2004 | 0.1 | 2004-2007 | 1.7 | 2007-2020 | 0.7* | 0.8* |
| Larynx | 1990-2020 | -0.9* |  |  |  |  |  |  |  |  |  |  | -0.9* |
| Lung | 1990-2012 | 2.5* | 2012-2016 | 1.2* | 2016-2020 | -0.7* |  |  |  |  |  |  | 1.9* |
| Bone | 1990-2014 | -2.3* | 2014-2020 | -0.5 |  |  |  |  |  |  |  |  | -2* |
| Connective and soft tissue | 1990-2003 | -0.3 | 2003-2020 | 1* |  |  |  |  |  |  |  |  | 0.4* |
| Skin, incl. melanoma | 1990-2015 | -0.1* | 2015-2020 | -2.5* |  |  |  |  |  |  |  |  | -0.5* |
| Breast | 1990-1995 | 0 | 1995-1998 | -2.2* | 1998-2014 | -1.5* | 2014-2018 | -0.3 | 2018-2020 | -2.4* |  |  | -1.2* |
| Uterus | 1990-2006 | -2.1* | 2006-2020 | -0.8* |  |  |  |  |  |  |  |  | -1.5* |
| Ovary | 1990-2011 | -1.1* | 2011-2020 | -1.4* |  |  |  |  |  |  |  |  | -1.2* |
| Bladder | 1990-2004 | -1.3* | 2004-2020 | -0.5* |  |  |  |  |  |  |  |  | -0.9* |
| Kidney | 1990-1994 | -0.1 | 1994-2007 | -1.5* | 2007-2016 | 0.1 | 2016-2020 | -1.8* |  |  |  |  | -0.9* |
| Thyroid | 1990-2007 | -3.2* | 2007-2020 | -2.1* |  |  |  |  |  |  |  |  | -2.7* |
| HL | 1990-1992 | 1.2 | 1992-2004 | -4.8* | 2004-2012 | -2.5* | 2012-2016 | -8.7* | 2016-2020 | -1.8 |  |  | -4* |
| NHL | 1990-1999 | 1.5* | 1999-2007 | -2.9* | 2007-2012 | -0.6 | 2012-2020 | -1.6* |  |  |  |  | -0.9* |
| MM | 1990-2001 | 1* | 2001-2020 | -1.6* |  |  |  |  |  |  |  |  | -0.6* |
| Leukemias | 1990-2002 | -1.2* | 2002-2018 | -1.6* | 2018-2020 | -4.1* |  |  |  |  |  |  | -1.6* |
| All neoplasms | 1990-1993 | 0 | 1993-2010 | -0.9* | 2010-2018 | -0.6* | 2018-2020 | -1.8* |  |  |  |  | -0.8* |
| **Females, 35-64 years** |  |  |  |  |  |  |  |  |  |  |  |  |  |
| Oral cavity and pharynx | 1990-2002 | 1.7* | 2002-2018 | -0.5* | 2018-2020 | -6.6* |  |  |  |  |  |  | 0 |
| Esophagus | 1990-2006 | 0.7* | 2006-2020 | -1.1* |  |  |  |  |  |  |  |  | -0.1 |
| Stomach | 1990-2005 | -3.2* | 2005-2020 | -2.1* |  |  |  |  |  |  |  |  | -2.7* |
| Colorectum | 1990-1993 | -0.5 | 1993-2020 | -1.5* |  |  |  |  |  |  |  |  | -1.4* |
| Liver | 1990-1997 | -0.6 | 1997-2000 | -12.9* | 2000-2020 | 1.3* |  |  |  |  |  |  | -0.7 |
| Gallbladder and bile ducts | 1990-1994 | -1.7* | 1994-2009 | -3.6* | 2009-2012 | -0.6 | 2012-2020 | -4* |  |  |  |  | -3.2* |
| Pancreas | 1990-2007 | 0.9* | 2007-2020 | 0.3* |  |  |  |  |  |  |  |  | 0.6* |
| Larynx | 1990-1993 | 3.8 | 1993-2000 | -1.6 | 2000-2003 | 5.2 | 2003-2010 | -3.5* | 2010-2014 | 0.6 | 2014-2020 | -5.2* | -1.3 |
| Lung | 1990-1997 | 2.6* | 1997-2007 | 3.5* | 2007-2015 | 0.9* | 2015-2020 | -3.2* |  |  |  |  | 1.5* |
| Bone | 1990-2020 | -2.7* |  |  |  |  |  |  |  |  |  |  | -2.7* |
| Connective and soft tissue | 1990-2000 | -0.2 | 2000-2020 | 0.9* |  |  |  |  |  |  |  |  | 0.5* |
| Skin, includ. melanoma | 1990-2013 | -0.1 | 2013-2020 | -3.5* |  |  |  |  |  |  |  |  | -0.9* |
| Breast | 1990-1995 | -0.3 | 1995-2015 | -2.2* | 2015-2018 | -0.1 | 2018-2020 | -3.2* |  |  |  |  | -1.8* |
| Uterus | 1990-2020 | -1.7* |  |  |  |  |  |  |  |  |  |  | -1.7* |
| Ovary | 1990-2020 | -1.7* |  |  |  |  |  |  |  |  |  |  | -1.7* |
| Bladder | 1990-1995 | -3.1* | 1995-2020 | 0.3* |  |  |  |  |  |  |  |  | -0.3 |
| Kidney | 1990-2008 | -2* | 2008-2017 | -0.6 | 2017-2020 | -4.8* |  |  |  |  |  |  | -1.9* |
| Thyroid | 1990-2020 | -3.3* |  |  |  |  |  |  |  |  |  |  | -3.3* |
| HL | 1990-1994 | -2.1 | 1994-2003 | -6.4* | 2003-2020 | -3.8* |  |  |  |  |  |  | -4.3* |
| NHL | 1990-2000 | 0.7* | 2000-2007 | -4.8* | 2007-2020 | -2.1* |  |  |  |  |  |  | -1.8* |
| MM | 1990-2002 | -0.1 | 2002-2020 | -2.7* |  |  |  |  |  |  |  |  | -1.7* |
| Leukemias | 1990-1996 | -0.8 | 1996-2020 | -2.4* |  |  |  |  |  |  |  |  | -2.1* |
| All neoplasms | 1990-1993 | -0.3 | 1993-2008 | -1* | 2008-2018 | -1.2* | 2018-2020 | -3* |  |  |  |  | -1.1* |

APC: annual percent change; AAPC: average annual percent change; HL: Hodgkin's lymphoma NHL: non-Hodgkin's lymphoma; MM: multiple myeloma.

*Significantly different from 0 (p<0.05).

# **Table S5**. Results of joinpoint analysis for mortality from all neoplasms in 23 selected European countries, among males and females of all ages and the 35-64 age group, from 1990 up to the most recent calendar year available.

| **Country, sex, age group** | **Trend 1** | | **Trend 2** | | **Trend 3** | | **Trend 4** | | **Trend 5** | | **Entire period** |
| --- | --- | --- | --- | --- | --- | --- | --- | --- | --- | --- | --- |
|  | **Years** | **APC** | **Years** | **APC** | **Years** | **APC** | **Years** | **APC** | **Years** | **APC** | **AAPC** |
|  |  |  |  |  |  |  |  |  |  |  |  |
| **Males, all ages** |  |  |  |  |  |  |  |  |  |  |  |
| Austria | 1990-2021 | -1.7* |  |  |  |  |  |  |  |  | -1.7* |
| Belarus | 1990-1995 | 1.8* | 1995-2009 | -1.1* | 2009-2013 | -2.5 | 2013-2018 | 0.7 |  |  | -0.5* |
| Belgium | 1990-1995 | -0.4 | 1995-2004 | -2.4* | 2004-2014 | -1.7* | 2014-2020 | -3.5* |  |  | -2* |
| Bulgaria | 1990-2003 | -0.1 | 2003-2006 | 3.9 | 2006-2021 | -0.9* |  |  |  |  | -0.1 |
| Czech Republic | 1990-2003 | -1.2* | 2003-2014 | -2.9* | 2014-2021 | -1.8* |  |  |  |  | -1.9* |
| Denmark | 1990-1999 | -0.6* | 1999-2021 | -1.9* |  |  |  |  |  |  | -1.5* |
| Finland | 1990-2008 | -1.8* | 2008-2021 | -1.2* |  |  |  |  |  |  | -1.5* |
| France | 1990-2002 | -1* | 2002-2020 | -2.2* |  |  |  |  |  |  | -1.7* |
| Germany | 1990-1993 | 0.3 | 1993-2006 | -2* | 2006-2020 | -1.3* |  |  |  |  | -1.4* |
| Greece | 1990-2000 | 0 | 2000-2011 | -0.9* | 2011-2014 | 2 | 2014-2020 | -1.3* |  |  | -0.4 |
| Hungary | 1990-1999 | 0.6* | 1999-2020 | -1.6* |  |  |  |  |  |  | -1* |
| Italy | 1990-2001 | -1.5* | 2001-2020 | -2* |  |  |  |  |  |  | -1.8* |
| Netherlands | 1990-1993 | -0.3 | 1993-2016 | -1.7* | 2016-2019 | -4.2* | 2019-2022 | -0.8 |  |  | -1.7* |
| Norway | 1990-1996 | 0.3 | 1996-2010 | -1.5* | 2010-2016 | -2.3* |  |  |  |  | -1.3* |
| Poland | 1990-2002 | 0.2 | 2002-2019 | -1.4* | 2019-2021 | -4.2* |  |  |  |  | -1* |
| Portugal | 1990-1996 | 0.8* | 1996-2006 | -0.6* | 2006-2019 | -0.2* |  |  |  |  | -0.1 |
| Romania | 1990-1993 | 3.6* | 1993-1999 | 0.6* | 1999-2002 | 2.3* | 2002-2016 | 0.4* | 2016-2019 | -2.4* | 0.7* |
| Serbia | 1998-2009 | 1.1* | 2009-2019 | -0.9* | 2019-2022 | -4.1* |  |  |  |  | -0.4* |
| Slovakia | 1992-1998 | 1.8* | 1998-2009 | -1.9* | 2009-2013 | 0.7 | 2013-2021 | -2.7* |  |  | -1* |
| Spain | 1990-1998 | 0.1 | 1998-2012 | -1.6* | 2012-2021 | -2.1* |  |  |  |  | -1.3* |
| Sweden | 1990-2003 | -0.7* | 2003-2017 | -1.6* | 2017-2022 | -3.1* |  |  |  |  | -1.5* |
| Switzerland | 1990-1997 | -2.7* | 1997-2015 | -1.8* | 2015-2020 | -3* |  |  |  |  | -2.2* |
| United Kingdom | 1990-1992 | -0.5 | 1992-1999 | -2.2* | 1999-2002 | -0.4 | 2002-2005 | -2.1 | 2005-2020 | -1.4* | -1.5* |
| **Males, 35-64 years** |  |  |  |  |  |  |  |  |  |  |  |
| Austria | 1990-2011 | -1.7* | 2011-2021 | -3.5* |  |  |  |  |  |  | -2.3* |
| Belarus | 1990-1994 | 2* | 1994-2013 | -1.8* | 2013-2018 | -0.3 |  |  |  |  | -1* |
| Belgium | 1990-1995 | -0.5 | 1995-2013 | -2.2* | 2013-2020 | -4.6* |  |  |  |  | -2.5* |
| Bulgaria | 1990-1997 | 1.4* | 1997-2000 | -4 | 2000-2007 | 1.7* | 2007-2021 | -2.2* |  |  | -0.7 |
| Czech Republic | 1990-2004 | -2.1* | 2004-2016 | -4* | 2016-2021 | -2.8* |  |  |  |  | -3* |
| Denmark | 1990-1997 | -0.7 | 1997-2016 | -2.4* | 2016-2021 | -5.1* |  |  |  |  | -2.4* |
| Finland | 1990-2021 | -1.9* |  |  |  |  |  |  |  |  | -1.9* |
| France | 1990-2002 | -1.5* | 2002-2009 | -2.2* | 2009-2017 | -3.1* | 2017-2020 | -4.8* |  |  | -2.4* |
| Germany | 1990-1992 | 1 | 1992-1999 | -2.5* | 1999-2016 | -1.9* | 2016-2020 | -3.2* |  |  | -2* |
| Greece | 1990-2010 | -0.7* | 2010-2013 | 1.8 | 2013-2020 | -1.9* |  |  |  |  | -0.7* |
| Hungary | 1990-1999 | 0.6* | 1999-2009 | -1.4* | 2009-2018 | -3.1* | 2018-2020 | -6* |  |  | -1.6* |
| Italy | 1990-2020 | -2.6* |  |  |  |  |  |  |  |  | -2.6* |
| Netherlands | 1990-2014 | -1.8* | 2014-2022 | -3.2* |  |  |  |  |  |  | -2.2* |
| Norway | 1990-1999 | -1.1* | 1999-2016 | -2.6* |  |  |  |  |  |  | -2.1* |
| Poland | 1990-2003 | -1.2* | 2003-2019 | -2.5* | 2019-2021 | -5.5* |  |  |  |  | -2.2* |
| Portugal | 1990-2019 | -0.2* |  |  |  |  |  |  |  |  | -0.2* |
| Romania | 1990-1993 | 3.4* | 1993-2007 | 0.4* | 2007-2017 | -0.6* | 2017-2019 | -5.3* |  |  | 0 |
| Serbia | 1998-2008 | 0.9* | 2008-2019 | -2* | 2019-2022 | -6* |  |  |  |  | -1.3* |
| Slovakia | 1992-1998 | 0 | 1998-2018 | -2.7* | 2018-2021 | -6.4* |  |  |  |  | -2.5* |
| Spain | 1990-1992 | 1.7 | 1992-1999 | -0.6* | 1999-2011 | -2* | 2011-2021 | -3.2* |  |  | -1.9* |
| Sweden | 1990-2014 | -1.8* | 2014-2022 | -3.5* |  |  |  |  |  |  | -2.3* |
| Switzerland | 1990-2010 | -2.2* | 2010-2020 | -3.5* |  |  |  |  |  |  | -2.6* |
| United Kingdom | 1990-2020 | -2* |  |  |  |  |  |  |  |  | -2* |
| **Females, all ages** |  |  |  |  |  |  |  |  |  |  |  |
| Austria | 1990-1993 | 0.1 | 1993-1998 | -2.5* | 1998-2009 | -1.1* | 2009-2012 | 0.3 | 2012-2021 | -1.7* | -1.3* |
| Belarus | 1990-1999 | -0.5* | 1999-2018 | -1.2* |  |  |  |  |  |  | -0.9* |
| Belgium | 1990-1995 | -0.3 | 1995-2004 | -1.5* | 2004-2011 | -0.2 | 2011-2020 | -1.9* |  |  | -1.1* |
| Bulgaria | 1990-1996 | 0.7 | 1996-2003 | -1.4* | 2003-2006 | 4.4 | 2006-2010 | -2.2 | 2010-2021 | 0 | -0.1 |
| Czech Republic | 1990-2003 | -0.9* | 2003-2013 | -2.2* | 2013-2021 | -1.1* |  |  |  |  | -1.4* |
| Denmark | 1990-1999 | -0.2 | 1999-2021 | -2* |  |  |  |  |  |  | -1.5* |
| Finland | 1990-2021 | -1* |  |  |  |  |  |  |  |  | -1* |
| France | 1990-2003 | -0.3* | 2003-2013 | -1.1* | 2013-2017 | 0 | 2017-2020 | -2* |  |  | -0.7* |
| Germany | 1990-1993 | -0.1 | 1993-2005 | -1.7* | 2005-2020 | -0.6* |  |  |  |  | -1* |
| Greece | 1990-2011 | -0.4* | 2011-2020 | 0.2 |  |  |  |  |  |  | -0.3* |
| Hungary | 1990-1999 | 0.3 | 1999-2006 | -1.7* | 2006-2014 | -0.1 | 2014-2020 | -1.3* |  |  | -0.6* |
| Italy | 1990-2020 | -1* |  |  |  |  |  |  |  |  | -1* |
| Netherlands | 1990-2011 | -0.4* | 2011-2022 | -1.6* |  |  |  |  |  |  | -0.8* |
| Norway | 1990-1996 | 1.1* | 1996-2016 | -1.3* |  |  |  |  |  |  | -0.8* |
| Poland | 1990-2003 | 0.1 | 2003-2011 | -0.8* | 2011-2019 | -0.2 | 2019-2021 | -4* |  |  | -0.5* |
| Portugal | 1990-2013 | -1* | 2013-2019 | 0.2 |  |  |  |  |  |  | -0.7* |
| Romania | 1990-2002 | 0.9* | 2002-2010 | -0.6* | 2010-2017 | 0.5 | 2017-2019 | -2.6 |  |  | 0.1 |
| Serbia | 1998-2009 | 0.8* | 2009-2022 | -0.5* |  |  |  |  |  |  | 0.1 |
| Slovakia | 1992-1998 | 1* | 1998-2010 | -1* | 2010-2014 | 1.5 | 2014-2021 | -1.8* |  |  | -0.4 |
| Spain | 1990-1994 | -0.3 | 1994-2007 | -1.2* | 2007-2011 | -0.3 | 2011-2021 | -0.9* |  |  | -0.9* |
| Sweden | 1990-2004 | -0.4* | 2004-2017 | -1.2* | 2017-2022 | -2.5* |  |  |  |  | -1.1* |
| Switzerland | 1990-1998 | -2.1* | 1998-2016 | -1* | 2016-2020 | -2.2* |  |  |  |  | -1.5* |
| United Kingdom | 1990-2020 | -1.2* |  |  |  |  |  |  |  |  | -1.2* |
| **Females, 35-64 years** |  |  |  |  |  |  |  |  |  |  |  |
| Austria | 1990-2015 | -1.4* | 2015-2021 | -3.2* |  |  |  |  |  |  | -1.8* |
| Belarus | 1990-1992 | 3.5 | 1992-2018 | -1.2* |  |  |  |  |  |  | -0.8* |
| Belgium | 1990-2013 | -1.2* | 2013-2020 | -3.2* |  |  |  |  |  |  | -1.7* |
| Bulgaria | 1990-2021 | -0.2* |  |  |  |  |  |  |  |  | -0.2* |
| Czech Republic | 1990-2003 | -1.5* | 2003-2021 | -2.4* |  |  |  |  |  |  | -2* |
| Denmark | 1990-1995 | -0.2 | 1995-2021 | -2.8* |  |  |  |  |  |  | -2.4* |
| Finland | 1990-2010 | -1.2* | 2010-2021 | -2.1* |  |  |  |  |  |  | -1.5* |
| France | 1990-2004 | -0.2 | 2004-2020 | -1.1* |  |  |  |  |  |  | -0.7* |
| Germany | 1990-1993 | -0.3 | 1993-1999 | -2* | 1999-2018 | -1.2* | 2018-2020 | -2.8* |  |  | -1.4* |
| Greece | 1990-2004 | -1.4* | 2004-2020 | 0.4* |  |  |  |  |  |  | -0.4* |
| Hungary | 1990-1999 | 0.5* | 1999-2014 | -0.7* | 2014-2020 | -2.8* |  |  |  |  | -0.8* |
| Italy | 1990-2020 | -1.2* |  |  |  |  |  |  |  |  | -1.2* |
| Netherlands | 1990-2011 | -0.5* | 2011-2022 | -2.9* |  |  |  |  |  |  | -1.3* |
| Norway | 1990-1997 | 0.8 | 1997-2016 | -2.5* |  |  |  |  |  |  | -1.6* |
| Poland | 1990-2007 | -0.4* | 2007-2019 | -1.6* | 2019-2021 | -5.4* |  |  |  |  | -1.2* |
| Portugal | 1990-2014 | -1.2* | 2014-2019 | 0.5 |  |  |  |  |  |  | -0.9* |
| Romania | 1990-2003 | 0.5* | 2003-2011 | -1.4* | 2011-2014 | 1.8 | 2014-2019 | -1.9* |  |  | -0.3 |
| Serbia | 1998-2008 | 0.6* | 2008-2022 | -0.9* |  |  |  |  |  |  | -0.3* |
| Slovakia | 1992-1999 | 0.2 | 1999-2019 | -1.2* | 2019-2021 | -6.2 |  |  |  |  | -1.2* |
| Spain | 1990-2005 | -1.3* | 2005-2013 | -0.3 | 2013-2021 | -1.4* |  |  |  |  | -1* |
| Sweden | 1990-2005 | -1* | 2005-2022 | -2.8* |  |  |  |  |  |  | -2* |
| Switzerland | 1990-2010 | -1.5* | 2010-2020 | -2.6* |  |  |  |  |  |  | -1.9* |
| United Kingdom | 1990-1999 | -2.3* | 1999-2020 | -1.7* |  |  |  |  |  |  | -1.9* |
|  |  |  |  |  |  |  |  |  |  |  |  |

APC: annual percent change; AAPC: average annual percent change.

*Significantly different from 0 (p<0.05).

# **Table S6**. Results of joinpoint analysis for mortality from colorectal cancer in 23 selected European countries, among males and females of all ages and the 35-64 age group, from 1990 up to the most recent calendar year available.

| **Country, sex, age group** | **Trend 1** | | **Trend 2** | | **Trend 3** | | **Trend 4** | | **Trend 5** | | **Entire period** |
| --- | --- | --- | --- | --- | --- | --- | --- | --- | --- | --- | --- |
|  | **Years** | **APC** | **Years** | **APC** | **Years** | **APC** | **Years** | **APC** | **Years** | **APC** | **AAPC** |
|  |  |  |  |  |  |  |  |  |  |  |  |
| **Males, all ages** |  |  |  |  |  |  |  |  |  |  |  |
| Austria | 1990-1993 | 2 | 1993-2012 | -3.1* | 2012-2021 | -1.8* |  |  |  |  | -2.2* |
| Belarus | 1990-1997 | 3.2* | 1997-2018 | -0.3 |  |  |  |  |  |  | 0.5* |
| Belgium | 1990-1994 | 1.6 | 1994-2015 | -1.9* | 2015-2020 | -4.6* |  |  |  |  | -1.9* |
| Bulgaria | 1990-2007 | 1.1* | 2007-2021 | 0.1 |  |  |  |  |  |  | 0.6* |
| Czech Republic | 1990-2003 | -0.7* | 2003-2014 | -4.3* | 2014-2021 | -2.2* |  |  |  |  | -2.3* |
| Denmark | 1990-1994 | 1 | 1994-2010 | -1.8* | 2010-2021 | -2.9* |  |  |  |  | -1.8* |
| Finland | 1990-2009 | -1* | 2009-2021 | 0.1 |  |  |  |  |  |  | -0.6* |
| France | 1990-2017 | -1.8* | 2017-2020 | -3.4* |  |  |  |  |  |  | -2* |
| Germany | 1990-1992 | 2.6 | 1992-2003 | -1.9* | 2003-2007 | -3.8* | 2007-2020 | -2* |  |  | -1.9* |
| Greece | 1990-1994 | 5* | 1994-2020 | 0.6* |  |  |  |  |  |  | 1.2* |
| Hungary | 1990-1999 | 1.6* | 1999-2020 | -0.7* |  |  |  |  |  |  | 0 |
| Italy | 1990-2004 | -0.8* | 2004-2020 | -2* |  |  |  |  |  |  | -1.4* |
| Netherlands | 1990-2010 | -0.2* | 2010-2017 | -2.1* | 2017-2020 | -7.1* | 2020-2022 | 1.8 |  |  | -1.2* |
| Norway | 1990-1992 | 5.5 | 1992-2016 | -1.3* |  |  |  |  |  |  | -0.8* |
| Poland | 1990-2001 | 2.7* | 2001-2015 | 0.4* | 2015-2021 | -1.8* |  |  |  |  | 0.7* |
| Portugal | 1990-1992 | -2.9 | 1992-1995 | 4.8 | 1995-2010 | 0.3* | 2010-2019 | -1.4* |  |  | 0 |
| Romania | 1990-2007 | 3.2* | 2007-2019 | 1.6* |  |  |  |  |  |  | 2.5* |
| Serbia | 1998-2010 | 1.8* | 2010-2022 | -0.8* |  |  |  |  |  |  | 0.5* |
| Slovakia | 1992-1999 | 4.1* | 1999-2021 | -1.5* |  |  |  |  |  |  | -0.2 |
| Spain | 1990-1995 | 2.5* | 1995-2012 | 0.2* | 2012-2021 | -2.4* |  |  |  |  | -0.2 |
| Sweden | 1990-2013 | -0.6* | 2013-2022 | -2.2* |  |  |  |  |  |  | -1.1* |
| Switzerland | 1990-2020 | -2.1* |  |  |  |  |  |  |  |  | -2.1* |
| United Kingdom | 1990-2014 | -1.6* | 2014-2020 | -0.5 |  |  |  |  |  |  | -1.4* |
| **Males, 35-64 years** |  |  |  |  |  |  |  |  |  |  |  |
| Austria | 1990-2021 | -3* |  |  |  |  |  |  |  |  | -3* |
| Belarus | 1990-1995 | 3.5 | 1995-2018 | -0.8* |  |  |  |  |  |  | 0 |
| Belgium | 1990-2020 | -1.9* |  |  |  |  |  |  |  |  | -1.9* |
| Bulgaria | 1990-2021 | -0.1 |  |  |  |  |  |  |  |  | -0.1 |
| Czech Republic | 1990-2004 | -1.3* | 2004-2015 | -5.3* | 2015-2021 | -1.4 |  |  |  |  | -2.8* |
| Denmark | 1990-1994 | 3 | 1994-2021 | -2.6* |  |  |  |  |  |  | -1.9* |
| Finland | 1990-2021 | -0.5* |  |  |  |  |  |  |  |  | -0.5* |
| France | 1990-2018 | -1.7* | 2018-2020 | -5 |  |  |  |  |  |  | -2* |
| Germany | 1990-1993 | 1.4 | 1993-2013 | -2.6* | 2013-2020 | -1.4* |  |  |  |  | -1.9* |
| Greece | 1990-2020 | 0.9* |  |  |  |  |  |  |  |  | 0.9* |
| Hungary | 1990-2004 | 0.9* | 2004-2020 | -0.9* |  |  |  |  |  |  | -0.1 |
| Italy | 1990-2002 | -1* | 2002-2020 | -2.2* |  |  |  |  |  |  | -1.7* |
| Netherlands | 1990-2017 | -0.8* | 2017-2022 | -4.6* |  |  |  |  |  |  | -1.4* |
| Norway | 1990-2016 | -1.9* |  |  |  |  |  |  |  |  | -1.9* |
| Poland | 1990-2001 | 0.8* | 2001-2015 | -0.3* | 2015-2021 | -1.7* |  |  |  |  | -0.2 |
| Portugal | 1990-1997 | 2.2* | 1997-2019 | -0.3* |  |  |  |  |  |  | 0.3 |
| Romania | 1990-2007 | 2.4* | 2007-2019 | 0.9* |  |  |  |  |  |  | 1.7* |
| Serbia | 1998-2005 | 2.7* | 2005-2022 | -0.7* |  |  |  |  |  |  | 0.3 |
| Slovakia | 1992-1999 | 2.5 | 1999-2021 | -2.4* |  |  |  |  |  |  | -1.2* |
| Spain | 1990-1994 | 2.9* | 1994-2012 | 0 | 2012-2021 | -2.9* |  |  |  |  | -0.5* |
| Sweden | 1990-2022 | -1* |  |  |  |  |  |  |  |  | -1* |
| Switzerland | 1990-2020 | -2* |  |  |  |  |  |  |  |  | -2* |
| United Kingdom | 1990-1992 | 2.4 | 1992-1999 | -3.3* | 1999-2014 | -1.7* | 2014-2020 | 1.2 |  |  | -1.2* |
| **Females, all ages** |  |  |  |  |  |  |  |  |  |  |  |
| Austria | 1990-2009 | -3.2* | 2009-2021 | -2* |  |  |  |  |  |  | -2.7* |
| Belarus | 1990-2018 | -0.5 |  |  |  |  |  |  |  |  | -0.5 |
| Belgium | 1990-1993 | 3.9 | 1993-2020 | -2.3* |  |  |  |  |  |  | -1.7* |
| Bulgaria | 1990-1996 | 1.5 | 1996-2003 | -2.3* | 2003-2008 | 3.4 | 2008-2011 | -6 | 2011-2021 | 0 | -0.3 |
| Czech Republic | 1990-2004 | -1.5* | 2004-2007 | -7.4* | 2007-2021 | -2.7* |  |  |  |  | -2.6* |
| Denmark | 1990-1999 | -0.5 | 1999-2021 | -2.4* |  |  |  |  |  |  | -1.8* |
| Finland | 1990-2021 | -0.9* |  |  |  |  |  |  |  |  | -0.9* |
| France | 1990-2020 | -1.7* |  |  |  |  |  |  |  |  | -1.7* |
| Germany | 1990-1993 | 0 | 1993-2010 | -3.3* | 2010-2020 | -1.9* |  |  |  |  | -2.5* |
| Greece | 1990-1996 | 2.4* | 1996-2020 | -0.7* |  |  |  |  |  |  | -0.1 |
| Hungary | 1990-2000 | 0.1 | 2000-2003 | -3.5 | 2003-2020 | -1.1* |  |  |  |  | -1* |
| Italy | 1990-2020 | -1.7* |  |  |  |  |  |  |  |  | -1.7* |
| Netherlands | 1990-2012 | -0.4* | 2012-2022 | -3.1* |  |  |  |  |  |  | -1.2* |
| Norway | 1990-1996 | 1.6 | 1996-2016 | -1.2* |  |  |  |  |  |  | -0.6 |
| Poland | 1990-2001 | 1.3* | 2001-2007 | -1.3* | 2007-2012 | 0.6 | 2012-2021 | -1.7* |  |  | -0.2 |
| Portugal | 1990-2019 | -0.9* |  |  |  |  |  |  |  |  | -0.9* |
| Romania | 1990-2006 | 1.8* | 2006-2019 | 0.1 |  |  |  |  |  |  | 1.1* |
| Serbia | 1998-2008 | 1.5* | 2008-2022 | -1.4* |  |  |  |  |  |  | -0.2 |
| Slovakia | 1992-1998 | 2.1 | 1998-2021 | -1.6* |  |  |  |  |  |  | -0.8* |
| Spain | 1990-1994 | 1.5* | 1994-2003 | -1* | 2003-2006 | -2.8 | 2006-2011 | 0.4 | 2011-2021 | -2.2* | -1* |
| Sweden | 1990-2022 | -0.7* |  |  |  |  |  |  |  |  | -0.7* |
| Switzerland | 1990-2020 | -1.7* |  |  |  |  |  |  |  |  | -1.7* |
| United Kingdom | 1990-2000 | -2.7* | 2000-2014 | -1* | 2014-2020 | 0.3 |  |  |  |  | -1.3* |
| **Females, 35-64 years** |  |  |  |  |  |  |  |  |  |  |  |
| Austria | 1990-2015 | -3.2* | 2015-2021 | 1.3 |  |  |  |  |  |  | -2.3* |
| Belarus | 1990-2018 | -0.8 |  |  |  |  |  |  |  |  | -0.8 |
| Belgium | 1990-2020 | -2* |  |  |  |  |  |  |  |  | -2* |
| Bulgaria | 1990-2021 | -1* |  |  |  |  |  |  |  |  | -1* |
| Czech Republic | 1990-2003 | -1.6* | 2003-2007 | -7.5* | 2007-2021 | -1.8* |  |  |  |  | -2.5* |
| Denmark | 1990-2021 | -2.3* |  |  |  |  |  |  |  |  | -2.3* |
| Finland | 1990-2021 | -0.5* |  |  |  |  |  |  |  |  | -0.5* |
| France | 1990-2010 | -1.5* | 2010-2020 | -0.7* |  |  |  |  |  |  | -1.2* |
| Germany | 1990-1993 | -1 | 1993-2005 | -3.4* | 2005-2013 | -2.1* | 2013-2020 | -0.6 |  |  | -2.2* |
| Greece | 1990-2020 | 0 |  |  |  |  |  |  |  |  | 0 |
| Hungary | 1990-2020 | -0.8* |  |  |  |  |  |  |  |  | -0.8* |
| Italy | 1990-2004 | -1.2* | 2004-2020 | -2.3* |  |  |  |  |  |  | -1.8* |
| Netherlands | 1990-2012 | -0.3 | 2012-2022 | -2.8* |  |  |  |  |  |  | -1.1* |
| Norway | 1990-2016 | -1.5* |  |  |  |  |  |  |  |  | -1.5* |
| Poland | 1990-2016 | -0.4* | 2016-2021 | -2.8* |  |  |  |  |  |  | -0.8* |
| Portugal | 1990-2019 | -0.8* |  |  |  |  |  |  |  |  | -0.8* |
| Romania | 1990-2019 | 0.5* |  |  |  |  |  |  |  |  | 0.5* |
| Serbia | 1998-2022 | -0.3 |  |  |  |  |  |  |  |  | -0.3 |
| Slovakia | 1992-2021 | -1.5* |  |  |  |  |  |  |  |  | -1.5* |
| Spain | 1990-2002 | -0.4 | 2002-2006 | -4.2* | 2006-2011 | 2.1 | 2011-2021 | -2.7* |  |  | -1.3* |
| Sweden | 1990-2022 | -0.8* |  |  |  |  |  |  |  |  | -0.8* |
| Switzerland | 1990-2020 | -1.3* |  |  |  |  |  |  |  |  | -1.3* |
| United Kingdom | 1990-2001 | -3.6* | 2001-2013 | -0.8* | 2013-2020 | 1.1 |  |  |  |  | -1.4* |
|  |  |  |  |  |  |  |  |  |  |  |  |

APC: annual percent change; AAPC: average annual percent change.

*Significantly different from 0 (p<0.05).

# **Table S7**. Results of joinpoint analysis for mortality from pancreatic cancer in 23 selected European countries, among males and females of all ages and the 35-64 age group, from 1990 up to the most recent calendar year available.

| **Country, sex, age group** | **Trend 1** | | **Trend 2** | | **Trend 3** | | **Trend 4** | | **Trend 5** | | **Trend 6** | | **Entire period** |
| --- | --- | --- | --- | --- | --- | --- | --- | --- | --- | --- | --- | --- | --- |
|  | **Years** | **APC** | **Years** | **APC** | **Years** | **APC** | **Years** | **APC** | **Years** | **APC** | **Years** | **APC** | **AAPC** |
|  |  |  |  |  |  |  |  |  |  |  |  |  |  |
| **Males, all ages** |  |  |  |  |  |  |  |  |  |  |  |  |  |
| Austria | 1990-2021 | -0.1 |  |  |  |  |  |  |  |  |  |  | -0.1 |
| Belarus | 2002-2018 | 1.2* |  |  |  |  |  |  |  |  |  |  | 1.2* |
| Belgium | 1990-2020 | -0.1 |  |  |  |  |  |  |  |  |  |  | -0.1 |
| Bulgaria | 1990-2021 | 1.1* |  |  |  |  |  |  |  |  |  |  | 1.1* |
| Czech Republic | 1990-2021 | -0.3* |  |  |  |  |  |  |  |  |  |  | -0.3* |
| Denmark | 1990-2021 | 0.2 |  |  |  |  |  |  |  |  |  |  | 0.2 |
| Finland | 1990-2021 | 0.2 |  |  |  |  |  |  |  |  |  |  | 0.2 |
| France | 1990-2020 | 0.4* |  |  |  |  |  |  |  |  |  |  | 0.4* |
| Germany | 1990-2020 | 0.3* |  |  |  |  |  |  |  |  |  |  | 0.3* |
| Greece | 1990-2020 | 1* |  |  |  |  |  |  |  |  |  |  | 1* |
| Hungary | 1990-2020 | 0.2* |  |  |  |  |  |  |  |  |  |  | 0.2* |
| Italy | 1990-2020 | 0.1* |  |  |  |  |  |  |  |  |  |  | 0.1* |
| Netherlands | 1990-1998 | -2.1* | 1998-2009 | 1.3* | 2009-2022 | -0.9* |  |  |  |  |  |  | -0.5 |
| Norway | 1990-2016 | -0.1 |  |  |  |  |  |  |  |  |  |  | -0.1 |
| Poland | 1990-2014 | -0.2* | 2014-2021 | -2.4* |  |  |  |  |  |  |  |  | -0.7* |
| Portugal | 1990-2004 | 0.1 | 2004-2019 | 1.8* |  |  |  |  |  |  |  |  | 1* |
| Romania | 1990-2008 | 2* | 2008-2019 | -0.2 |  |  |  |  |  |  |  |  | 1.2* |
| Serbia | 1998-2009 | 2* | 2009-2022 | 0.1 |  |  |  |  |  |  |  |  | 0.9* |
| Slovakia | 1992-2021 | 0.2 |  |  |  |  |  |  |  |  |  |  | 0.2 |
| Spain | 1990-2021 | 0.8* |  |  |  |  |  |  |  |  |  |  | 0.8* |
| Sweden | 1990-2022 | -0.1 |  |  |  |  |  |  |  |  |  |  | -0.1 |
| Switzerland | 1990-1999 | -1.8* | 1999-2020 | 0.4* |  |  |  |  |  |  |  |  | -0.2 |
| United Kingdom | 1990-1995 | -2.9* | 1995-2020 | 0.2* |  |  |  |  |  |  |  |  | -0.3* |
| **Males, 35-64 years** |  |  |  |  |  |  |  |  |  |  |  |  |  |
| Austria | 1990-2021 | -0.5* |  |  |  |  |  |  |  |  |  |  | -0.5* |
| Belarus | 2002-2018 | 1.1* |  |  |  |  |  |  |  |  |  |  | 1.1* |
| Belgium | 1990-2020 | -0.4* |  |  |  |  |  |  |  |  |  |  | -0.4* |
| Bulgaria | 1990-2021 | 0.5* |  |  |  |  |  |  |  |  |  |  | 0.5* |
| Czech Republic | 1990-2021 | -1* |  |  |  |  |  |  |  |  |  |  | -1* |
| Denmark | 1990-2021 | -0.6* |  |  |  |  |  |  |  |  |  |  | -0.6* |
| Finland | 1990-2021 | -0.2 |  |  |  |  |  |  |  |  |  |  | -0.2 |
| France | 1990-2007 | 0.4* | 2007-2020 | -0.7* |  |  |  |  |  |  |  |  | -0.1 |
| Germany | 1990-2020 | -0.3* |  |  |  |  |  |  |  |  |  |  | -0.3* |
| Greece | 1990-2020 | 1.1* |  |  |  |  |  |  |  |  |  |  | 1.1* |
| Hungary | 1990-2020 | 0 |  |  |  |  |  |  |  |  |  |  | 0 |
| Italy | 1990-2006 | -0.1 | 2006-2015 | -1.2* | 2015-2020 | 0.7 |  |  |  |  |  |  | -0.3 |
| Netherlands | 1990-2014 | 0.1 | 2014-2022 | -2.6* |  |  |  |  |  |  |  |  | -0.6* |
| Norway | 1990-2016 | -0.4 |  |  |  |  |  |  |  |  |  |  | -0.4 |
| Poland | 1990-2009 | -0.3* | 2009-2021 | -2.1* |  |  |  |  |  |  |  |  | -1* |
| Portugal | 1990-2019 | 0.8* |  |  |  |  |  |  |  |  |  |  | 0.8* |
| Romania | 1990-1997 | 3.3* | 1997-2014 | 0.8* | 2014-2019 | -2 |  |  |  |  |  |  | 0.9* |
| Serbia | 1998-2022 | 0.2 |  |  |  |  |  |  |  |  |  |  | 0.2 |
| Slovakia | 1992-2021 | -0.4* |  |  |  |  |  |  |  |  |  |  | -0.4* |
| Spain | 1990-2002 | 1.4* | 2002-2021 | 0 |  |  |  |  |  |  |  |  | 0.5* |
| Sweden | 1990-2022 | -0.8* |  |  |  |  |  |  |  |  |  |  | -0.8* |
| Switzerland | 1990-2020 | -0.3 |  |  |  |  |  |  |  |  |  |  | -0.3 |
| United Kingdom | 1990-2020 | -0.4* |  |  |  |  |  |  |  |  |  |  | -0.4* |
| **Females, all ages** |  |  |  |  |  |  |  |  |  |  |  |  |  |
| Austria | 1990-2021 | 0.3* |  |  |  |  |  |  |  |  |  |  | 0.3* |
| Belarus | 2002-2018 | 1.1 |  |  |  |  |  |  |  |  |  |  | 1.1 |
| Belgium | 1990-2020 | 0.7* |  |  |  |  |  |  |  |  |  |  | 0.7* |
| Bulgaria | 1990-2021 | 1.7* |  |  |  |  |  |  |  |  |  |  | 1.7* |
| Czech Republic | 1990-2021 | 0.2* |  |  |  |  |  |  |  |  |  |  | 0.2* |
| Denmark | 1990-2021 | 0 |  |  |  |  |  |  |  |  |  |  | 0 |
| Finland | 1990-1995 | -3.1 | 1995-2021 | 0.6* |  |  |  |  |  |  |  |  | 0 |
| France | 1990-2020 | 1.4* |  |  |  |  |  |  |  |  |  |  | 1.4* |
| Germany | 1990-2020 | 0.8* |  |  |  |  |  |  |  |  |  |  | 0.8* |
| Greece | 1990-1999 | 2.3* | 1999-2004 | -1.3 | 2004-2007 | 5.8 | 2007-2011 | -1.3 | 2011-2015 | 4.4* | 2015-2020 | -0.7 | 1.3* |
| Hungary | 1990-2020 | 0.7* |  |  |  |  |  |  |  |  |  |  | 0.7* |
| Italy | 1990-2020 | 0.8* |  |  |  |  |  |  |  |  |  |  | 0.8* |
| Netherlands | 1990-2011 | 0.8* | 2011-2022 | -0.1 |  |  |  |  |  |  |  |  | 0.5* |
| Norway | 1990-2016 | 0.2 |  |  |  |  |  |  |  |  |  |  | 0.2 |
| Poland | 1990-2014 | 0.2* | 2014-2021 | -1.2* |  |  |  |  |  |  |  |  | -0.1 |
| Portugal | 1990-2019 | 1.1* |  |  |  |  |  |  |  |  |  |  | 1.1* |
| Romania | 1990-2009 | 2.1* | 2009-2019 | 0.8* |  |  |  |  |  |  |  |  | 1.7* |
| Serbia | 1998-2022 | 1.3* |  |  |  |  |  |  |  |  |  |  | 1.3* |
| Slovakia | 1992-2021 | 1.1* |  |  |  |  |  |  |  |  |  |  | 1.1* |
| Spain | 1990-2021 | 1.4* |  |  |  |  |  |  |  |  |  |  | 1.4* |
| Sweden | 1990-2022 | -0.1 |  |  |  |  |  |  |  |  |  |  | -0.1 |
| Switzerland | 1990-1996 | -2 | 1996-2020 | 0.8* |  |  |  |  |  |  |  |  | 0.2 |
| United Kingdom | 1990-1996 | -1.5* | 1996-2020 | 0.5* |  |  |  |  |  |  |  |  | 0.1 |
| **Females, 35-64 years** |  |  |  |  |  |  |  |  |  |  |  |  |  |
| Austria | 1990-2021 | 0 |  |  |  |  |  |  |  |  |  |  | 0 |
| Belarus | 2002-2018 | -0.1 |  |  |  |  |  |  |  |  |  |  | -0.1 |
| Belgium | 1990-2020 | 0.7* |  |  |  |  |  |  |  |  |  |  | 0.7* |
| Bulgaria | 1990-2021 | 1.5* |  |  |  |  |  |  |  |  |  |  | 1.5* |
| Czech Republic | 1990-2021 | -0.3 |  |  |  |  |  |  |  |  |  |  | -0.3 |
| Denmark | 1990-2021 | -0.9* |  |  |  |  |  |  |  |  |  |  | -0.9* |
| Finland | 1990-2021 | -0.1 |  |  |  |  |  |  |  |  |  |  | -0.1 |
| France | 1990-2020 | 1.5* |  |  |  |  |  |  |  |  |  |  | 1.5* |
| Germany | 1990-2020 | 0.6* |  |  |  |  |  |  |  |  |  |  | 0.6* |
| Greece | 1990-2004 | 0.9 | 2004-2007 | 11 | 2007-2010 | -10.7 | 2010-2013 | 13.4 | 2013-2020 | -1 |  |  | 1.4 |
| Hungary | 1990-2020 | 0.7* |  |  |  |  |  |  |  |  |  |  | 0.7* |
| Italy | 1990-2020 | 0.6* |  |  |  |  |  |  |  |  |  |  | 0.6* |
| Netherlands | 1990-2022 | 0.2 |  |  |  |  |  |  |  |  |  |  | 0.2 |
| Norway | 1990-2016 | -0.1 |  |  |  |  |  |  |  |  |  |  | -0.1 |
| Poland | 1990-2014 | -0.1 | 2014-2021 | -1.9* |  |  |  |  |  |  |  |  | -0.5* |
| Portugal | 1990-2019 | 1.4* |  |  |  |  |  |  |  |  |  |  | 1.4* |
| Romania | 1990-1994 | 5.7* | 1994-2019 | 0.7* |  |  |  |  |  |  |  |  | 1.4* |
| Serbia | 1998-2022 | 1* |  |  |  |  |  |  |  |  |  |  | 1* |
| Slovakia | 1992-2021 | 0.8* |  |  |  |  |  |  |  |  |  |  | 0.8* |
| Spain | 1990-2021 | 1.5* |  |  |  |  |  |  |  |  |  |  | 1.5* |
| Sweden | 1990-2022 | -0.9* |  |  |  |  |  |  |  |  |  |  | -0.9* |
| Switzerland | 1990-2020 | 0 |  |  |  |  |  |  |  |  |  |  | 0 |
| United Kingdom | 1990-1996 | -2.5* | 1996-2020 | 0.2 |  |  |  |  |  |  |  |  | -0.4 |
|  |  |  |  |  |  |  |  |  |  |  |  |  |  |

APC: annual percent change; AAPC: average annual percent change.

*Significantly different from 0 (p<0.05).

# **Table S8**. Results of joinpoint analysis for mortality from lung cancer in 23 selected European countries, among males and females of all ages and the 35-64 age group, from 1990 up to the most recent calendar year available.

| **Country, sex, age group** | **Trend 1** | | **Trend 2** | | **Trend 3** | | **Trend 4** | | **Trend 5** | | **Trend 6** | | **Entire period** |
| --- | --- | --- | --- | --- | --- | --- | --- | --- | --- | --- | --- | --- | --- |
|  | **Years** | **APC** | **Years** | **APC** | **Years** | **APC** | **Years** | **APC** | **Years** | **APC** | **Years** | **APC** | **AAPC** |
|  |  |  |  |  |  |  |  |  |  |  |  |  |  |
| **Males, all ages** |  |  |  |  |  |  |  |  |  |  |  |  |  |
| Austria | 1990-2021 | -2.1* |  |  |  |  |  |  |  |  |  |  | -2.1* |
| Belarus | 1990-1995 | 2.1* | 1995-2009 | -1.9* | 2009-2013 | -4 | 2013-2018 | 0 |  |  |  |  | -1.2* |
| Belgium | 1990-1995 | -0.5 | 1995-2000 | -4.1* | 2000-2011 | -2.1* | 2011-2020 | -5.1* |  |  |  |  | -3.1* |
| Bulgaria | 1990-1994 | 2.3 | 1994-2000 | -2.3* | 2000-2008 | 2.3* | 2008-2021 | -1.8* |  |  |  |  | -0.3 |
| Czech Republic | 1990-2005 | -2.6* | 2005-2021 | -4* |  |  |  |  |  |  |  |  | -3.3* |
| Denmark | 1990-2014 | -2.1* | 2014-2021 | -5* |  |  |  |  |  |  |  |  | -2.8* |
| Finland | 1990-2001 | -3.5* | 2001-2021 | -2.5* |  |  |  |  |  |  |  |  | -2.8* |
| France | 1990-1998 | -0.2 | 1998-2007 | -1.1* | 2007-2015 | -1.9* | 2015-2020 | -4.3* |  |  |  |  | -1.6* |
| Germany | 1990-1993 | -0.3 | 1993-2010 | -2.2* | 2010-2014 | -0.7 | 2014-2020 | -2.9* |  |  |  |  | -2* |
| Greece | 1990-1994 | 0.5 | 1994-2011 | -0.6* | 2011-2014 | 2.4 | 2014-2020 | -2.7* |  |  |  |  | -0.6* |
| Hungary | 1990-1998 | 0.6 | 1998-2017 | -1.6* | 2017-2020 | -5.6* |  |  |  |  |  |  | -1.4* |
| Italy | 1990-1992 | -0.3 | 1992-1999 | -2.2* | 1999-2008 | -2.8* | 2008-2011 | -2* | 2011-2018 | -3.1* | 2018-2020 | -4.6* | -2.6* |
| Netherlands | 1990-2011 | -2.7* | 2011-2022 | -4.2* |  |  |  |  |  |  |  |  | -3.2* |
| Norway | 1990-2002 | -0.1 | 2002-2010 | -1.5* | 2010-2016 | -3.7* |  |  |  |  |  |  | -1.4* |
| Poland | 1990-2004 | -0.6* | 2004-2017 | -2.7* | 2017-2021 | -6.1* |  |  |  |  |  |  | -2.2* |
| Portugal | 1990-2019 | 0.3* |  |  |  |  |  |  |  |  |  |  | 0.3* |
| Romania | 1990-1994 | 4* | 1994-2015 | 0.4* | 2015-2019 | -3.4* |  |  |  |  |  |  | 0.3* |
| Serbia | 1998-2008 | 2* | 2008-2019 | -1.1* | 2019-2022 | -8.5* |  |  |  |  |  |  | -0.8* |
| Slovakia | 1992-1995 | 1.7 | 1995-2018 | -2.6* | 2018-2021 | -6.8* |  |  |  |  |  |  | -2.6* |
| Spain | 1990-1995 | 1.2* | 1995-2010 | -1.1* | 2010-2021 | -2.6* |  |  |  |  |  |  | -1.3* |
| Sweden | 1990-2011 | -1.3* | 2011-2022 | -4.4* |  |  |  |  |  |  |  |  | -2.4* |
| Switzerland | 1990-2020 | -2.6* |  |  |  |  |  |  |  |  |  |  | -2.6* |
| United Kingdom | 1990-1999 | -3.9* | 1999-2004 | -3* | 2004-2008 | -1.5* | 2008-2018 | -2.7* | 2018-2020 | -5.3* |  |  | -3.1* |
| **Males, 35-64 years** |  |  |  |  |  |  |  |  |  |  |  |  |  |
| Austria | 1990-2010 | -1.9* | 2010-2021 | -4.3* |  |  |  |  |  |  |  |  | -2.7* |
| Belarus | 1990-1993 | 3.3 | 1993-2018 | -2.7* |  |  |  |  |  |  |  |  | -2* |
| Belgium | 1990-2012 | -2.6* | 2012-2020 | -7.1* |  |  |  |  |  |  |  |  | -3.8* |
| Bulgaria | 1990-1995 | 1.8 | 1995-2000 | -3.1* | 2000-2008 | 1.5* | 2008-2021 | -3.4* |  |  |  |  | -1.3* |
| Czech Republic | 1990-2007 | -3.9* | 2007-2021 | -6.7* |  |  |  |  |  |  |  |  | -5.2* |
| Denmark | 1990-2014 | -2.9* | 2014-2021 | -7.1* |  |  |  |  |  |  |  |  | -3.9* |
| Finland | 1990-2001 | -4.5* | 2001-2010 | -1.5* | 2010-2021 | -4.5* |  |  |  |  |  |  | -3.6* |
| France | 1990-2005 | -0.5* | 2005-2014 | -2.3* | 2014-2020 | -5.9* |  |  |  |  |  |  | -2.1* |
| Germany | 1990-2001 | -2.6* | 2001-2016 | -2.1* | 2016-2020 | -5.5* |  |  |  |  |  |  | -2.7* |
| Greece | 1990-2000 | 0.1 | 2000-2014 | -0.7* | 2014-2020 | -4.4* |  |  |  |  |  |  | -1.2* |
| Hungary | 1990-1998 | 0.5 | 1998-2006 | -2.2* | 2006-2009 | 1.1 | 2009-2018 | -4.3* | 2018-2020 | -10.4* |  |  | -2.4* |
| Italy | 1990-2012 | -3.8* | 2012-2020 | -4.7* |  |  |  |  |  |  |  |  | -4* |
| Netherlands | 1990-1993 | -0.3 | 1993-2001 | -4.4* | 2001-2012 | -2.4* | 2012-2022 | -4.7* |  |  |  |  | -3.4* |
| Norway | 1990-2010 | -1.9* | 2010-2016 | -6.7* |  |  |  |  |  |  |  |  | -3* |
| Poland | 1990-2004 | -2* | 2004-2015 | -3.7* | 2015-2021 | -6.7* |  |  |  |  |  |  | -3.5* |
| Portugal | 1990-2013 | 0.4* | 2013-2019 | -2* |  |  |  |  |  |  |  |  | -0.1 |
| Romania | 1990-1994 | 3.5* | 1994-2015 | -0.8* | 2015-2019 | -4.5* |  |  |  |  |  |  | -0.7* |
| Serbia | 1998-2008 | 1.9* | 2008-2018 | -2.3* | 2018-2022 | -10.4* |  |  |  |  |  |  | -2* |
| Slovakia | 1992-2013 | -3.8* | 2013-2021 | -6.5* |  |  |  |  |  |  |  |  | -4.5* |
| Spain | 1990-1999 | 0.2 | 1999-2010 | -1.5* | 2010-2021 | -4.4* |  |  |  |  |  |  | -2* |
| Sweden | 1990-2011 | -2.6* | 2011-2022 | -7.2* |  |  |  |  |  |  |  |  | -4.2* |
| Switzerland | 1990-2020 | -3.3* |  |  |  |  |  |  |  |  |  |  | -3.3* |
| United Kingdom | 1990-1997 | -4.9* | 1997-2016 | -2.9* | 2016-2020 | -5.2* |  |  |  |  |  |  | -3.6* |
| **Females, all ages** |  |  |  |  |  |  |  |  |  |  |  |  |  |
| Austria | 1990-2015 | 2* | 2015-2021 | -0.1 |  |  |  |  |  |  |  |  | 1.6* |
| Belarus | 1990-2002 | -2.7* | 2002-2018 | -0.7 |  |  |  |  |  |  |  |  | -1.6* |
| Belgium | 1990-2002 | 2* | 2002-2011 | 4* | 2011-2020 | -0.9* |  |  |  |  |  |  | 1.7* |
| Bulgaria | 1990-2002 | -0.1 | 2002-2021 | 2.3* |  |  |  |  |  |  |  |  | 1.4* |
| Czech Republic | 1990-1999 | 2.5* | 1999-2012 | 0.9* | 2012-2021 | -1* |  |  |  |  |  |  | 0.8* |
| Denmark | 1990-2006 | 1* | 2006-2021 | -2.2* |  |  |  |  |  |  |  |  | -0.5* |
| Finland | 1990-2014 | 1.9* | 2014-2021 | -0.6 |  |  |  |  |  |  |  |  | 1.3* |
| France | 1990-2006 | 4.5* | 2006-2014 | 2.8* | 2014-2020 | -0.2 |  |  |  |  |  |  | 3.1* |
| Germany | 1990-2014 | 2.7* | 2014-2020 | -0.3 |  |  |  |  |  |  |  |  | 2.1* |
| Greece | 1990-2006 | 0.3 | 2006-2013 | 4.2* | 2013-2020 | 1.6* |  |  |  |  |  |  | 1.5* |
| Hungary | 1990-1996 | 3.9* | 1996-2015 | 2.2* | 2015-2020 | -1.8* |  |  |  |  |  |  | 1.9* |
| Italy | 1990-2017 | 1.5* | 2017-2020 | -1.1 |  |  |  |  |  |  |  |  | 1.3* |
| Netherlands | 1990-1993 | 7.4* | 1993-2007 | 4* | 2007-2014 | 0.7 | 2014-2022 | -1.9* |  |  |  |  | 2.1* |
| Norway | 1990-2004 | 3.5* | 2004-2016 | -0.1 |  |  |  |  |  |  |  |  | 1.8* |
| Poland | 1990-2015 | 2.5* | 2015-2021 | -1.6* |  |  |  |  |  |  |  |  | 1.7* |
| Portugal | 1990-1997 | -0.3 | 1997-2019 | 2.4* |  |  |  |  |  |  |  |  | 1.8* |
| Romania | 1990-2019 | 1.9* |  |  |  |  |  |  |  |  |  |  | 1.9* |
| Serbia | 1998-2009 | 4.5* | 2009-2019 | 1.9* | 2019-2022 | -4.2* |  |  |  |  |  |  | 2.3* |
| Slovakia | 1992-2016 | 1.9* | 2016-2021 | -1.3 |  |  |  |  |  |  |  |  | 1.3* |
| Spain | 1990-1996 | 1.6* | 1996-2013 | 4.5* | 2013-2021 | 2.2* |  |  |  |  |  |  | 3.4* |
| Sweden | 1990-2006 | 2.7* | 2006-2017 | -1* | 2017-2022 | -3.9* |  |  |  |  |  |  | 0.4* |
| Switzerland | 1990-2010 | 2.5* | 2010-2020 | -0.6 |  |  |  |  |  |  |  |  | 1.4* |
| United Kingdom | 1990-2003 | -0.6* | 2003-2008 | 1.3* | 2008-2015 | -0.7* | 2015-2020 | -2.6* |  |  |  |  | -0.6* |
| **Females, 35-64 years** |  |  |  |  |  |  |  |  |  |  |  |  |  |
| Austria | 1990-2010 | 2.8* | 2010-2021 | -2.3* |  |  |  |  |  |  |  |  | 1* |
| Belarus | 1990-2018 | -1.5* |  |  |  |  |  |  |  |  |  |  | -1.5* |
| Belgium | 1990-2002 | 2.1* | 2002-2005 | 7.8 | 2005-2013 | 1.8* | 2013-2020 | -4.1* |  |  |  |  | 1.1 |
| Bulgaria | 1990-2021 | 2.1* |  |  |  |  |  |  |  |  |  |  | 2.1* |
| Czech Republic | 1990-2006 | 1.4* | 2006-2018 | -1.9* | 2018-2021 | -9.3* |  |  |  |  |  |  | -1* |
| Denmark | 1990-2010 | -0.9* | 2010-2021 | -4.4* |  |  |  |  |  |  |  |  | -2.1* |
| Finland | 1990-2008 | 2.5* | 2008-2021 | -1.9* |  |  |  |  |  |  |  |  | 0.6* |
| France | 1990-2006 | 6* | 2006-2014 | 2.5* | 2014-2020 | -2.1* |  |  |  |  |  |  | 3.4* |
| Germany | 1990-2007 | 3.7* | 2007-2015 | 0.8* | 2015-2020 | -3.5* |  |  |  |  |  |  | 1.7* |
| Greece | 1990-1993 | -5.9 | 1993-2004 | 1.1 | 2004-2013 | 4.6* | 2013-2020 | -0.7 |  |  |  |  | 1 |
| Hungary | 1990-2000 | 4.3* | 2000-2005 | 1.1 | 2005-2008 | 6.5 | 2008-2015 | -0.1 | 2015-2020 | -5* |  |  | 1.3* |
| Italy | 1990-1997 | 0.6 | 1997-2012 | 2.3* | 2012-2020 | -1.1* |  |  |  |  |  |  | 1* |
| Netherlands | 1990-2007 | 3.6* | 2007-2014 | -0.5 | 2014-2022 | -4.8* |  |  |  |  |  |  | 0.6* |
| Norway | 1990-2008 | 1.4* | 2008-2016 | -4.2* |  |  |  |  |  |  |  |  | -0.4 |
| Poland | 1990-2009 | 3* | 2009-2017 | -1* | 2017-2021 | -7.6* |  |  |  |  |  |  | 0.6* |
| Portugal | 1990-2019 | 2.7* |  |  |  |  |  |  |  |  |  |  | 2.7* |
| Romania | 1990-2019 | 1.7* |  |  |  |  |  |  |  |  |  |  | 1.7* |
| Serbia | 1998-2009 | 4.9* | 2009-2018 | 1.1* | 2018-2022 | -5.3* |  |  |  |  |  |  | 1.7* |
| Slovakia | 1992-2016 | 1.8* | 2016-2021 | -6.1* |  |  |  |  |  |  |  |  | 0.4 |
| Spain | 1990-2010 | 5.9* | 2010-2017 | 2.6* | 2017-2021 | -2.6* |  |  |  |  |  |  | 4* |
| Sweden | 1990-2005 | 1.9* | 2005-2018 | -3.9* | 2018-2022 | -11.5* |  |  |  |  |  |  | -2.2* |
| Switzerland | 1990-1993 | 8.4 | 1993-2010 | 1.7* | 2010-2020 | -3.9* |  |  |  |  |  |  | 0.4 |
| United Kingdom | 1990-1997 | -2.5* | 1997-2010 | 0.1 | 2010-2020 | -2.9* |  |  |  |  |  |  | -1.5* |
|  |  |  |  |  |  |  |  |  |  |  |  |  |  |

APC: annual percent change; AAPC: average annual percent change.

*Significantly different from 0 (p<0.05).

# **Table S9**. Results of joinpoint analysis for mortality from breast cancer in 23 selected European countries among females of all ages and the 35-64 age group, from 1990 up to the most recent calendar year available.

| **Country, sex, age group** | **Trend 1** | | **Trend 2** | | **Trend 3** | | **Trend 4** | | **Entire period** |
| --- | --- | --- | --- | --- | --- | --- | --- | --- | --- |
|  | **Years** | **APC** | **Years** | **APC** | **Years** | **APC** | **Years** | **Years** | **APC** |
|  |  |  |  |  |  |  |  |  |  |
| **Females, all ages** |  |  |  |  |  |  |  |  |  |
| Austria | 1990-1994 | -0.8 | 1994-2011 | -2.4* | 2011-2021 | -1* |  |  | -1.7* |
| Belarus | 1990-2000 | 1.2* | 2000-2018 | -1.5* |  |  |  |  | -0.5 |
| Belgium | 1990-1995 | -0.2 | 1995-2011 | -2.1* | 2011-2020 | -3.3* |  |  | -2.1* |
| Bulgaria | 1990-2021 | -0.2* |  |  |  |  |  |  | -0.2* |
| Czech Republic | 1990-2005 | -1.5* | 2005-2009 | -6.6* | 2009-2021 | -0.8* |  |  | -1.9* |
| Denmark | 1990-1997 | 0.4 | 1997-2021 | -3.1* |  |  |  |  | -2.3* |
| Finland | 1990-2021 | -1.3* |  |  |  |  |  |  | -1.3* |
| France | 1990-1999 | -0.2 | 1999-2015 | -1.7* | 2015-2018 | 0.3 | 2018-2020 | -3.8* | -1.2* |
| Germany | 1990-1993 | 0.9 | 1993-2007 | -2* | 2007-2020 | -1* |  |  | -1.3* |
| Greece | 1990-2020 | -0.5* |  |  |  |  |  |  | -0.5* |
| Hungary | 1990-2000 | 0 | 2000-2006 | -3.5* | 2006-2020 | -0.8* |  |  | -1.1* |
| Italy | 1990-2014 | -1.6* | 2014-2020 | -0.2 |  |  |  |  | -1.3* |
| Netherlands | 1990-1997 | -0.9 | 1997-2022 | -2.5* |  |  |  |  | -2.1* |
| Norway | 1990-1994 | 1.8 | 1994-2016 | -2.7* |  |  |  |  | -2* |
| Poland | 1990-2010 | -0.7* | 2010-2019 | 0.8* | 2019-2021 | -4.9 |  |  | -0.5* |
| Portugal | 1990-2006 | -1.9* | 2006-2019 | -0.8* |  |  |  |  | -1.4* |
| Romania | 1990-2002 | 1* | 2002-2011 | -1.1* | 2011-2017 | 1.1 | 2017-2019 | -3.9 | 0 |
| Serbia | 1998-2022 | -0.2* |  |  |  |  |  |  | -0.2* |
| Slovakia | 1992-1999 | 1.7* | 1999-2009 | -2.3* | 2009-2017 | 2* | 2017-2021 | -3 | -0.3 |
| Spain | 1990-1993 | 1.3 | 1993-2004 | -2.6* | 2004-2021 | -1.6* |  |  | -1.7* |
| Sweden | 1990-2005 | -1.2* | 2005-2022 | -2.4* |  |  |  |  | -1.8* |
| Switzerland | 1990-1999 | -3.4* | 1999-2020 | -2* |  |  |  |  | -2.5* |
| United Kingdom | 1990-1999 | -2.8* | 1999-2014 | -2.4* | 2014-2020 | -1.4* |  |  | -2.3* |
| United Kingdom |  |  |  |  |  |  |  |  |  |
| **Females, 35-64 years** |  |  |  |  |  |  |  |  |  |
| Austria | 1990-2021 | -2.7* |  |  |  |  |  |  | -2.7* |
| Belarus | 1990-2007 | -0.1 | 2007-2018 | -3.3* |  |  |  |  | -1.4* |
| Belgium | 1990-1996 | -0.1 | 1996-2020 | -3.3* |  |  |  |  | -2.7* |
| Bulgaria | 1990-2021 | -0.8* |  |  |  |  |  |  | -0.8* |
| Czech Republic | 1990-1994 | -0.2 | 1994-2014 | -3.7* | 2014-2021 | 0.5 |  |  | -2.3* |
| Denmark | 1990-1996 | 0 | 1996-2021 | -4.1* |  |  |  |  | -3.3* |
| Finland | 1990-2021 | -2* |  |  |  |  |  |  | -2* |
| France | 1990-1999 | -0.2 | 1999-2015 | -2.4* | 2015-2020 | -0.5 |  |  | -1.4* |
| Germany | 1990-1995 | -0.3 | 1995-2010 | -2.8* | 2010-2020 | -1.6* |  |  | -2* |
| Greece | 1990-2020 | -1.3* |  |  |  |  |  |  | -1.3* |
| Hungary | 1990-2000 | -0.1 | 2000-2006 | -3.9* | 2006-2020 | -1.7* |  |  | -1.6* |
| Italy | 1990-2015 | -2* | 2015-2020 | -0.1 |  |  |  |  | -1.7* |
| Netherlands | 1990-1999 | -1.2* | 1999-2022 | -3* |  |  |  |  | -2.5* |
| Norway | 1990-1996 | 1.1 | 1996-2016 | -3.5* |  |  |  |  | -2.5* |
| Poland | 1990-2021 | -1* |  |  |  |  |  |  | -1* |
| Portugal | 1990-2019 | -1.9* |  |  |  |  |  |  | -1.9* |
| Romania | 1990-1999 | 0.8 | 1999-2019 | -1.1* |  |  |  |  | -0.5* |
| Serbia | 1998-2022 | -1.4* |  |  |  |  |  |  | -1.4* |
| Slovakia | 1992-2001 | 0 | 2001-2008 | -3.5* | 2008-2021 | -0.5 |  |  | -1.1* |
| Spain | 1990-1994 | 0.5 | 1994-2000 | -4.4* | 2000-2021 | -1.8* |  |  | -2.1* |
| Sweden | 1990-2005 | -1.6* | 2005-2022 | -3.4* |  |  |  |  | -2.6* |
| Switzerland | 1990-2020 | -3.1* |  |  |  |  |  |  | -3.1* |
| United Kingdom | 1990-2014 | -2.9* | 2014-2020 | -1.5* |  |  |  |  | -2.6* |
|  |  |  |  |  |  |  |  |  |  |

APC: annual percent change; AAPC: average annual percent change.

*Significantly different from 0 (p<0.05).

# **Table S10**. Results of joinpoint analysis for mortality from prostate cancer in 23 selected European countries, among males of all ages and the 35-64 age group, from 1990 up to the most recent calendar year available.

| **Country, sex, age group** | **Trend 1** | | **Trend 2** | | **Trend 3** | | **Trend 4** | | **Trend 5** | | **Entire period** |
| --- | --- | --- | --- | --- | --- | --- | --- | --- | --- | --- | --- |
|  | **Years** | **APC** | **Years** | **APC** | **Years** | **APC** | **Years** | **Years** | **APC** | **Years** | **APC** |
|  |  |  |  |  |  |  |  |  |  |  |  |
| **Males, all ages** |  |  |  |  |  |  |  |  |  |  |  |
| Austria | 1990-1999 | -0.5 | 1999-2013 | -3.6* | 2013-2021 | -0.1 |  |  |  |  | -1.8* |
| Belarus | 1990-2007 | 3.3* | 2007-2018 | 0 |  |  |  |  |  |  | 2* |
| Belgium | 1990-1995 | 1.9 | 1995-2007 | -4.6* | 2007-2020 | -1.9* |  |  |  |  | -2.4* |
| Bulgaria | 1990-2021 | 1.2* |  |  |  |  |  |  |  |  | 1.2* |
| Czech Republic | 1990-2004 | 1.4* | 2004-2007 | -7.7 | 2007-2021 | -1.9* |  |  |  |  | -1* |
| Denmark | 1990-2002 | 0.5 | 2002-2021 | -1.8* |  |  |  |  |  |  | -0.9* |
| Finland | 1990-1998 | 0.2 | 1998-2021 | -2.6* |  |  |  |  |  |  | -1.9* |
| France | 1990-2003 | -1.3* | 2003-2013 | -4.1* | 2013-2020 | -1.9* |  |  |  |  | -2.4* |
| Germany | 1990-1994 | 2.1* | 1994-2007 | -2.9* | 2007-2020 | -0.8* |  |  |  |  | -1.3* |
| Greece | 1990-2005 | 1.5* | 2005-2011 | -4* | 2011-2020 | -0.8* |  |  |  |  | -0.3 |
| Hungary | 1990-2000 | 1.4* | 2000-2005 | -6.4* | 2005-2020 | -0.2 |  |  |  |  | -0.8* |
| Italy | 1990-2003 | -1.1* | 2003-2014 | -3.2* | 2014-2020 | -1 |  |  |  |  | -1.9* |
| Netherlands | 1990-1995 | 1.2 | 1995-2022 | -2.2* |  |  |  |  |  |  | -1.7* |
| Norway | 1990-1996 | 2.2* | 1996-2016 | -2.4* |  |  |  |  |  |  | -1.3* |
| Poland | 1990-2002 | 3* | 2002-2013 | -0.8* | 2013-2016 | 4.1 | 2016-2021 | -0.9 |  |  | 1.1* |
| Portugal | 1990-1997 | 2.6* | 1997-2019 | -2.1* |  |  |  |  |  |  | -1* |
| Romania | 1990-1996 | 2.9* | 1996-2019 | 0.9* |  |  |  |  |  |  | 1.3* |
| Serbia | 1998-2010 | 3* | 2010-2022 | -1.5* |  |  |  |  |  |  | 0.7* |
| Slovakia | 1992-1998 | 6.6* | 1998-2009 | -1.4* | 2009-2014 | 4.2* | 2014-2021 | -2.6* |  |  | 0.9* |
| Spain | 1990-1998 | 0.7* | 1998-2008 | -3.7* | 2008-2011 | 0.2 | 2011-2015 | -4* | 2015-2021 | -1.7* | -1.9* |
| Sweden | 1990-2003 | 0.1 | 2003-2017 | -2.7* | 2017-2022 | -4.6* |  |  |  |  | -1.9* |
| Switzerland | 1990-2020 | -2.7* |  |  |  |  |  |  |  |  | -2.7* |
| United Kingdom | 1990-1992 | 2.8 | 1992-2000 | -1.7* | 2000-2003 | 1 | 2003-2006 | -3.5 | 2006-2020 | -1.1* | -1* |
| **Males, 35-64 years** |  |  |  |  |  |  |  |  |  |  |  |
| Austria | 1990-2021 | -2.5* |  |  |  |  |  |  |  |  | -2.5* |
| Belarus | 1990-2011 | 2.7* | 2011-2018 | -4.2* |  |  |  |  |  |  | 0.9 |
| Belgium | 1990-2020 | -2.9* |  |  |  |  |  |  |  |  | -2.9* |
| Bulgaria | 1990-2004 | 2.3* | 2004-2021 | -1.6* |  |  |  |  |  |  | 0.2 |
| Czech Republic | 1990-2004 | -0.2 | 2004-2021 | -2.8* |  |  |  |  |  |  | -1.6* |
| Denmark | 1990-2002 | -0.3 | 2002-2021 | -3.9* |  |  |  |  |  |  | -2.6* |
| Finland | 1990-2021 | -2.6* |  |  |  |  |  |  |  |  | -2.6* |
| France | 1990-2002 | -1.2* | 2002-2020 | -3.1* |  |  |  |  |  |  | -2.3* |
| Germany | 1990-1992 | 7.7 | 1992-2015 | -1.4* | 2015-2020 | -3.8* |  |  |  |  | -1.2* |
| Greece | 1990-2020 | -1.1* |  |  |  |  |  |  |  |  | -1.1* |
| Hungary | 1990-2020 | -0.8* |  |  |  |  |  |  |  |  | -0.8* |
| Italy | 1990-2020 | -2.3* |  |  |  |  |  |  |  |  | -2.3* |
| Netherlands | 1990-2006 | -0.8* | 2006-2022 | -2.7* |  |  |  |  |  |  | -1.7* |
| Norway | 1990-1998 | 2 | 1998-2016 | -4.8* |  |  |  |  |  |  | -2.8* |
| Poland | 1990-2019 | 0.1 | 2019-2021 | -10.1 |  |  |  |  |  |  | -0.6 |
| Portugal | 1990-2019 | -1.6* |  |  |  |  |  |  |  |  | -1.6* |
| Romania | 1990-2019 | 0.5* |  |  |  |  |  |  |  |  | 0.5* |
| Serbia | 1998-2022 | -0.3 |  |  |  |  |  |  |  |  | -0.3 |
| Slovakia | 1992-2021 | -0.5 |  |  |  |  |  |  |  |  | -0.5 |
| Spain | 1990-1994 | 2 | 1994-2021 | -2.4* |  |  |  |  |  |  | -1.8* |
| Sweden | 1990-2008 | -2.2* | 2008-2022 | -5* |  |  |  |  |  |  | -3.5* |
| Switzerland | 1990-2020 | -2.7* |  |  |  |  |  |  |  |  | -2.7* |
| United Kingdom | 1990-2020 | -1.6* |  |  |  |  |  |  |  |  | -1.6* |

APC: annual percent change; AAPC: average annual percent change.

*Significantly different from 0 (p<0.05).

# **Figure S1.** Age-standardized mortality rates from colorectal cancer per 100,000 males and females in 33 European countries and the EU-27 in 2020^a^.

^a^ Available year for Portugal and Romania: 2019; for Belarus: 2018; for Malta: 2017; for Norway: 2016.

# **Figure S2.** Age-standardized mortality rates from pancreatic cancer per 100,000 males and females in 33 European countries and the EU-27 in 2020^a^.

^^

^a^ Available year for Portugal and Romania: 2019; for Belarus: 2018; for Malta: 2017; for Norway: 2016.

# **Figure S3.** Age-standardized mortality rates from lung cancer per 100,000 males and females in 33 European countries and the EU-27 in 2020^a^.

^^

^a^ Available year for Portugal and Romania: 2019; for Belarus: 2018; for Malta: 2017; for Norway: 2016.

# **Figure S4.** Age-standardized mortality rates from breast cancer per 100,000 females in 33 European countries and the EU-27 in 2020^a^.

^a^ Available year for Portugal and Romania: 2019; for Belarus: 2018; for Malta: 2017; for Norway: 2016.

# **Figure S5.** Age-standardized mortality rates from prostate cancer per 100,000 males in 33 European countries and the EU-27 in 2020^a^.

^a^ Available year for Portugal and Romania: 2019; for Belarus: 2018; for Malta: 2017; for Norway: 2016.
